# Co_13_O_8_—metalloxocubes: a new class of perovskite-like neutral clusters with cubic aromaticity

**DOI:** 10.1093/nsr/nwaa201

**Published:** 2020-08-29

**Authors:** Lijun Geng, Mouyi Weng, Cong-Qiao Xu, Hanyu Zhang, Chaonan Cui, Haiming Wu, Xin Chen, Mingyu Hu, Hai Lin, Zhen-Dong Sun, Xi Wang, Han-Shi Hu, Jun Li, Jiaxin Zheng, Zhixun Luo, Feng Pan, Jiannian Yao

**Affiliations:** Beijing National Laboratory for Molecular Sciences (BNLMS), State Key Laboratory for Structural Chemistry of Unstable and Stable Species, Institute of Chemistry, Chinese Academy of Sciences, Beijing 100190, China; School of Physics, Shandong University, Jinan 250100, China; School of Advanced Materials, Peking University Shenzhen Graduate School, Shenzhen 518055, China; Department of Chemistry, Southern University of Science and Technology, Shenzhen 518055, China; Beijing National Laboratory for Molecular Sciences (BNLMS), State Key Laboratory for Structural Chemistry of Unstable and Stable Species, Institute of Chemistry, Chinese Academy of Sciences, Beijing 100190, China; University of Chinese Academy of Sciences, Beijing 100049, China; Beijing National Laboratory for Molecular Sciences (BNLMS), State Key Laboratory for Structural Chemistry of Unstable and Stable Species, Institute of Chemistry, Chinese Academy of Sciences, Beijing 100190, China; Beijing National Laboratory for Molecular Sciences (BNLMS), State Key Laboratory for Structural Chemistry of Unstable and Stable Species, Institute of Chemistry, Chinese Academy of Sciences, Beijing 100190, China; School of Advanced Materials, Peking University Shenzhen Graduate School, Shenzhen 518055, China; School of Advanced Materials, Peking University Shenzhen Graduate School, Shenzhen 518055, China; School of Advanced Materials, Peking University Shenzhen Graduate School, Shenzhen 518055, China; School of Physics, Shandong University, Jinan 250100, China; School of Physics and Electrical Engineering, Kashi University, Kashgar 844006, China; College of Science, Beijing Jiaotong University, Beijing 100044, China; Department of Chemistry and Key Laboratory of Organic Optoelectronics and Molecular Engineering of Ministry of Education, Tsinghua University, Beijing 100084, China; Department of Chemistry, Southern University of Science and Technology, Shenzhen 518055, China; Department of Chemistry and Key Laboratory of Organic Optoelectronics and Molecular Engineering of Ministry of Education, Tsinghua University, Beijing 100084, China; School of Advanced Materials, Peking University Shenzhen Graduate School, Shenzhen 518055, China; Beijing National Laboratory for Molecular Sciences (BNLMS), State Key Laboratory for Structural Chemistry of Unstable and Stable Species, Institute of Chemistry, Chinese Academy of Sciences, Beijing 100190, China; University of Chinese Academy of Sciences, Beijing 100049, China; School of Advanced Materials, Peking University Shenzhen Graduate School, Shenzhen 518055, China; Beijing National Laboratory for Molecular Sciences (BNLMS), State Key Laboratory for Structural Chemistry of Unstable and Stable Species, Institute of Chemistry, Chinese Academy of Sciences, Beijing 100190, China; School of Advanced Materials, Peking University Shenzhen Graduate School, Shenzhen 518055, China; University of Chinese Academy of Sciences, Beijing 100049, China

**Keywords:** metalloxocube, oxygen-passivated metal cluster, cubic aromaticity, cluster materials, magnetic property

## Abstract

Exploring stable clusters to understand structural evolution from atoms to macroscopic matter and to construct new materials is interesting yet challenging in chemistry. Utilizing our newly developed deep-ultraviolet laser ionization mass spectrometry technique, here we observe the reactions of neutral cobalt clusters with oxygen and find a very stable cluster species of Co_13_O_8_ that dominates the mass distribution in the presence of a large flow rate of oxygen gas. The results of global-minimum structural search reveal a unique cubic structure and distinctive stability of the neutral Co_13_O_8_ cluster that forms a new class of metal oxides that we named as ‘metalloxocubes’. Thermodynamics and kinetics calculations illustrate the structural evolution from icosahedral Co_13_ to the metalloxocube Co_13_O_8_ with decreased energy, enhanced stability and aromaticity. This class of neutral oxygen-passivated metal clusters may be an ideal candidate for genetic materials because of the cubic nature of the building blocks and the stability due to cubic aromaticity.

## INTRODUCTION

Building materials with well-defined components and stable structures is one of the foremost challenges in chemistry and cluster science. Extensive efforts have been made to explore new clusters with highly symmetrical regular structures, and occasional success has been achieved, such as the discovery of fullerene C_60_ [1], Au_20_ [2], etc. [3,4]. In particular, a few ‘magic’ metal clusters possessing special stability, such as Al_13_^−^ [5], have been found to be inert toward oxygen reactions due to the coincident closure of both electronic and geometric shells [6–8], embodying the nearly free electron gas (NFEG) theory of metals and epitomizing the jellium model of clusters within a symmetric potential function [9]. In view of this, the reactivity of metal clusters toward oxygen is often studied to explore the stability of these materials in the gas phase [10]. However, not all clusters are subject to this fundamental constraint. In some cases, cluster stability can be associated with aromaticity or superatom property [8,11,12], a large HOMO (highest occupied molecular orbital)–LUMO (lowest unoccupied molecular orbital) energy gap and a large spin excitation energy [13]. In particular, the metal cluster stability can be reinforced by ligand protection. In recent years, various ligand-protected metal clusters have been synthesized via wet chemistry [14–18], where diverse thiols are employed as stabilizers and ligands, allowing for electron transfer and hence balanced charge distribution, occasionally exhibiting superatom characteristics of the core [11,19].

For gas-phase naked metal clusters, especially those of main group elements, a few previous studies have illustrated that cluster stability can be altered by doping hydrogen or halogen ligands, which may induce formation of active sites on the cluster surface or passivate the metal cluster [20,21]. There are ongoing efforts devoted to exploring stable clusters for new materials, and to understanding atomically precise reactivity of metals. For example, Kapiloff and Ervin [22] studied the reactivity of small cobalt cluster anions Co*_n_*^−^ (*n* = 2–8) with O_2_, and noted rapid rate coefficients allowing fragmentation of the clusters (with a removal of CoO_2_^−^) independent of the cluster size. Bernstein and coworkers [23] studied 118-nm laser ionization of neutral cobalt oxides Co*_n_*O*_m_* (*n* = 2–11) formed by laser ablation in plasma atmosphere, and have identified a series of Co*_n_*O*_m_* clusters showing size-dependent mass abundances. Gutsev *et al.* [24] performed an systematic investigation on small (FeO)*_n_*, (CoO)*_n_* and (NiO)*_n_* clusters, where the 3d-metal oxides preferred oxygen bridge-linked Co(III) sites. Riley and coworkers [25] studied the reactions of cobalt clusters with water and ammonia, and found addition reactions to be dominant channels. Besides, Andersson *et al.* [26] studied the reactivities of neutral Fe, Co and Cu clusters at single-collision conditions and determined their reaction probability in a single collision. Among these studies, few-collision conditions are convenient to study size-selective cluster reactivity, and sufficient collision conditions are necessarily important to probe stable species in the gas phase. However, the reactivity and reaction-determined stability of preformed cobalt clusters and oxides, especially neutrals, have not been fully unveiled so far.

**Scheme 1.**
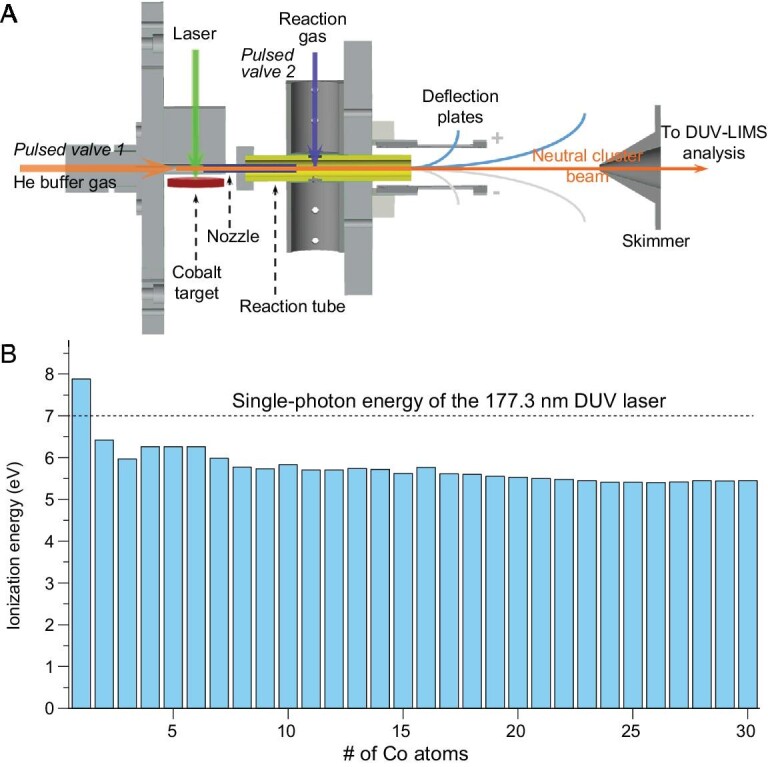
Instrumentation. (A) A sketch of instrument showing the sampling, reaction and deep-ultraviolet laser ionization mass spectrometry (DUV-LIMS) strategy. (B) Ionization energies of the neutral Co*_n_* (*n* = 1–30) clusters, which are <7 eV (except Co atom) suggesting unique advantages of the ps-pulsed 177.3-nm deep-ultraviolet laser available for single-photon ionization of these clusters. The values are from refs [28–30].

Recently, we have developed a highly sensitive mass spectrometer combined with a homemade ps-pulsed 177.3-nm (7 eV single-photon energy) deep-ultraviolet laser that has unique advantages of low fragmentation and high ionization efficiency, enabling detailed studies of neutral metal clusters that had been largely unexplored previously. Taking advantages of our optimized cluster source, flow tube reactor and the 177.3-nm laser (Scheme 1), here we are able to prepare and observe well-resolved Co*_n_* (*n* ≤ 30) clusters and provide insights into their reactivities. Interestingly, a very stable cluster species Co_13_O_8_ emerges in the mass spectra of neutral cobalt clusters reacting with oxygen, and shows dominant mass abundance in the presence of sufficient oxygen. Using first-principles theoretical calculations based on genetic algorithm and basin-hopping strategies, we find a cubic structure and distinctive stability of this neutral cluster Co_13_O_8_ and show that it possesses unique cubic aromaticity. This new class of metal oxides with a cubic structure and special aromatic stability is named as ‘metalloxocubes’. Such neutral oxygen-passivated metal clusters with high stability and cubic aromaticity help to expand the chemistry of ligand-protected metal clusters [27], and provide an ideal candidate for genetic materials [19] with a perovskite-like structure.

## RESULTS AND DISCUSSION

**Figure 1.**
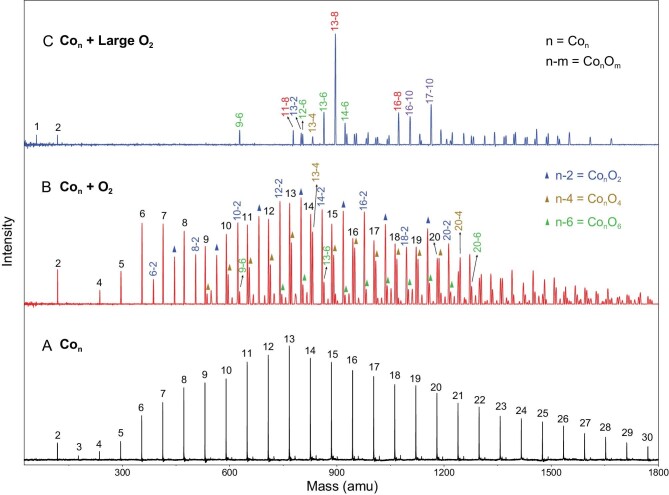
Mass spectrometry observation. (A) Representative size distribution of the naked Co*_n_* clusters. (B and C) The mass spectra after reaction of the Co*_n_* clusters with different amounts of 20% O_2_/He introduced into the flow tube (200 and 250 μs) and with varying pulse widths controlled by the pulse valve (more details in the Supplementary data). The Co*_n_* and Co*_n_*O*_m_* clusters are ionized by a ps-pulsed deep-ultraviolet 177.3-nm laser.

We prepared well-resolved neutral Co*_n_* clusters (*n* = 2–30) via a homemade laser evaporation (LaVa) source, with a typical distribution shown in Fig. 1A. In general, the preparation of pure neutral metal clusters with >10 metal atoms is challenging in view of the much lower bond energy for metal–metal bonds than for the metal–nonmetal bonds. Here, the ionization energies of all the Co*_n_* clusters (*n* = 2–30) are slightly smaller than 7 eV (Scheme 1); thus, the 177.3-nm laser happens to be a perfect ionization condition for such neutral Co*_n_* clusters. Single-photon ionization is available for high-efficiency ionization with absence of photoinduced fragmentation. Figure 1B presents a typical mass spectrum of the neutral Co*_n_* clusters upon reacting with oxygen (for details see Fig. S2).

As seen, all the nascent Co*_n_* clusters have decreased mass abundances; simultaneously, several Co*_n_*O*_m_* (*n* ≥ 6) products, typically seen as Co*_n_*O_2_, Co*_n_*O_4_ and Co*_n_*O_6_, appear in the spectrum. With an atomic electron structure of [Ar]3d^7^4s^2^, cobalt readily forms oxides, as the Co–O bond energy (∼3.99 eV) is larger than the Co–Co bond energy (∼1.73 eV) [30]. With the rapid growth into Co*_n_*O*_m_* (*m* ≥ 2), it is notable that the oxide products mostly bear an even number of oxygen atoms, indicating that the reactions are initiated by oxygen adsorption and activation on the cobalt clusters. This result is consistent with the previous findings [25,26]. Among the observed Co*_n_*O*_m_* clusters, the dominant peaks belong to the Co*_n_*O_2_ series, such as Co_12_O_2_, Co_13_O_2_ and Co_16_O_2_. A small portion of Co*_n_*O_4_ and Co*_n_*O_6_ clusters (such as Co_13_O_4_ and Co_13_O_6_) emerges in the mass spectra (Figs S3 and S4). This observation indicates that the Co*_n_* clusters could primarily follow a sequential reaction channel to form

the Co*_n_*O*_m_* products successively, i.e.

(1) \begin{eqnarray*} {\rm{C}}{{\rm{o}}_n} + x{{\rm{O}}_2} &\to& {\rm{C}}{{\rm{o}}_n}{{\rm{O}}_2} \to {\rm{C}}{{\rm{o}}_n}{{\rm{O}}_4} \to {\rm{C}}{{\rm{o}}_n}{{\rm{O}}_6}\nonumber\\ &\to& {\rm{C}}{{\rm{o}}_n}{{\rm{O}}_8}.\end{eqnarray*}

Upon further increasing the reactant oxygen content, however, we found that not all the Co*_n_* clusters continued developing into Co*_n_*O_8_ and Co*_n_*O_10_, as shown in Fig. 1C. Interestingly, the neutral Co_13_O_8_ dominates the mass distribution, showing its distinction in surviving the large flow rate oxygen etching reaction. Meanwhile, the small cobalt clusters could find chances to grow up via a relatively slow subsequent reaction channel under sufficient He-assisted three-body collisions, written as

(2) \begin{equation*}{\rm{C}}{{\rm{o}}_n}{{\rm{O}}_x} + {\rm{C}}{{\rm{o}}_m} + {\rm{He}} \to {\rm{C}}{{\rm{o}}_{n + m}}{{\rm{O}}_x} + {\rm{He}}.\end{equation*}

Note that, besides our calculations on Co_12–14_O_2–8_ clusters, previous studies indicated that all the Co*_n_*O_2_ (*n* > 3) and Co*_n_*O_4_ (*n* > 7) have an ionization energy <7 eV (for details see Tables S10 and S11). We have also prepared well-resolved Co*_n_*^±^, Fe*_n_*^+^ and Ni*_n_*^+^ clusters and observed their reactions with oxygen; as a result, enhanced stabilities of such a class of M_13_O_8_^±,0^ (M = Fe, Co, Ni) clusters were repeatedly observed (for details see Figs S6–S8). While this observation is consistent with a few previously identified stable species via gas-phase collisional reactions, such as Al_13_^−^ [5,31], Al_13_I^−^ [5], Al_13_I_2_^−^ [32] and Ag_13_^−^ [13], it is a puzzle why such a oxide cluster (Co_13_O_8_) with significantly low oxidation state can be stable.

The stability and reactivity of metal clusters are often associated with the nature of both the metal itself and the correlative oxide products, and magic metal clusters could be conclusively determined by O_2_ etching reactions [33,34]. Here, the DFT (density functional theory)-calculated binding energies and HOMO–LUMO gaps of Co*_n_* cluster do not find Co_13_ as a magic cluster (Fig. S12). According to the chemical valence of cobalt, the Co*_n_* clusters may follow an etching reaction toward the formation of typical bivalent and trivalent cobalt oxide molecules:

(3) \begin{eqnarray*} &&{\rm{C}}{{\rm{o}}_n} + {{\rm{O}}_2} \to 2{\rm{CoO}} + {\rm{C}}{{\rm{o}}_{n - 2}},\nonumber\\ && {\rm{C}}{{\rm{o}}_n} + 3{{\rm{O}}_2} \to 2{\rm{C}}{{\rm{o}}_2}{{\rm{O}}_3} + {\rm{C}}{{\rm{o}}_{n - 4}}.\end{eqnarray*}

On the other hand, the Co*_n_* clusters and their oxides Co*_n_*O*_m_* could undergo successive reactions with oxygen to generate stable species such as Co_13_O_8_. Based on these reaction channels, it is reasonable to yield distinctive Co_13_O_8_ clusters; however, it is unclear why Co_13_O_8_ is ‘magic’ and what kind of structure it is.

**Figure 2.**
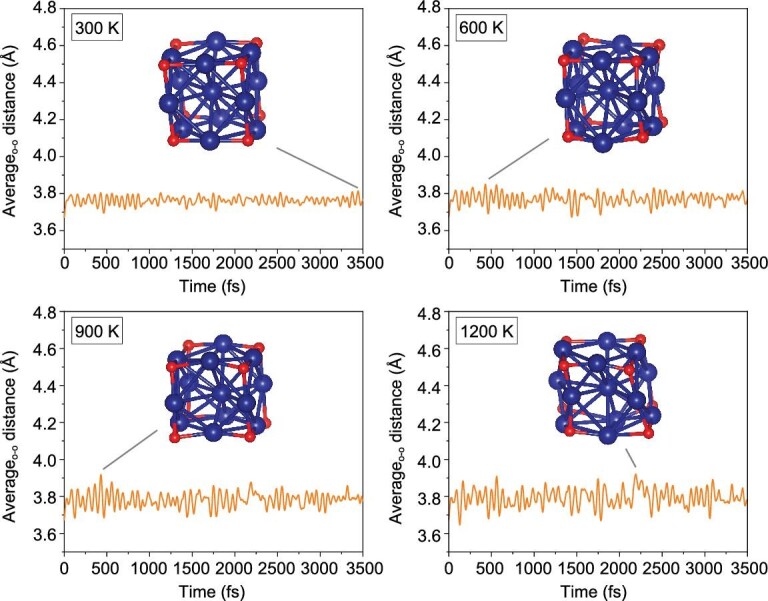
AIMD simulations of Co_13_O_8_ at 300, 600, 900 and 1200 K for 3500 fs, with the average oxygen–oxygen distance on the eight edges indicated in Å.

To determine the structure of Co_13_O_8_, we have conducted first-principles calculations based on a genetic algorithm strategy. The global lowest energy structures of all the Co*_n_* (3 ≤ *n* ≤ 16) and Co_13_O*_m_* (*m* = 2, 4, 6, 8) clusters are provided in Fig. S10. While bare Co_13_ could have an icosahedral structure [35,36], it is interesting to find that Co_13_O_8_ exhibits a body-centered cubic structure (a large HOMO–LUMO gap of 2.14 eV) with 12 cobalt atoms surrounding the inner core and 8 oxygen atoms coherently anchoring the 8 triangular facets of the Co@Co_12_. Such a cubic structure is consistent with the previously predicted structures of Fe_13_O_8_ [37,38]. It is usual that such a class of M_13_O_8_ clusters can survive sufficient oxygen etching reactions. In general, sufficient reactions in high-pressure gas collision cells tend to screen out stable clusters of highly degenerate energy states and spherically symmetric structures, such as the previous findings of Al_13_I_2_*_n_*^−^ [6], where the doping of iodine that is stretched out of an Al_13_ icosahedron helps to balance the surface charge enabling enhanced stability. Also, we did independent basin-hopping search for Co_13_O_8_ structure using TGMin code [39], and reproduced the pseudo-O_h_ global minimum of Co_13_O_8_. Further, we conducted *ab* *initio* molecular dynamics (AIMD) simulations to identify their relative stability. AIMD simulations indicate that Co_13_O_8_ has outstanding thermal stability, with the cubic structure undissociated even up to 1200 K, as shown in Fig. 2.

**Figure 3.**
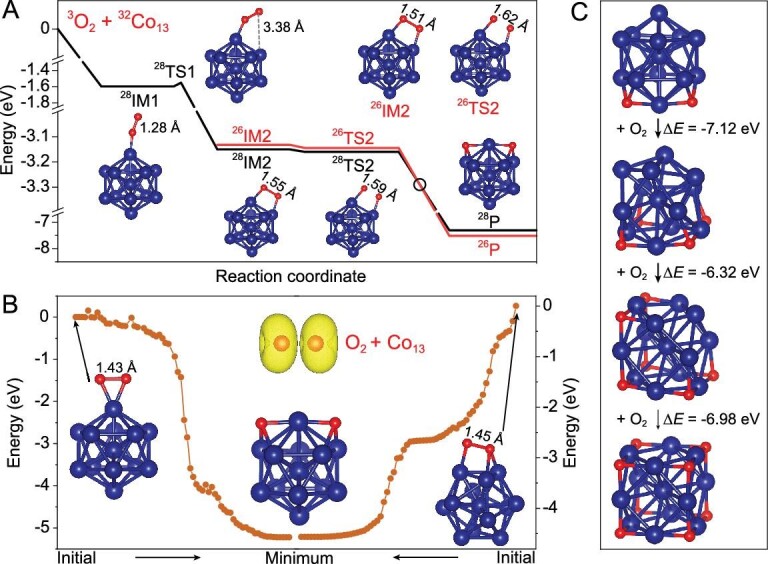
Chemical reaction dynamics. (A) The reaction coordinate for ‘O_2_ + Co_13_ → Co_13_O_2_’ via an ‘end-on’ coordination orientation, showing readily O–O bond dissociation on Co_13_. The black versus red lines correspond to spin crossing. (B) The dynamic optimization processes for ‘O_2_ + Co_13_’ via ‘side-on’ and ‘face-on’ attack orientations, showing spontaneous O–O bond dissociation of Co_13_·O_2_ toward the energy minimum. (C) Thermodynamic energy changes from Co_13_O_2_ to Co_13_O_8_ [Δ*E* = *E* (Co_13_O_2_*_x_*_+2_) – *E* (Co_13_O_2_*_x_*) – *E* (O_2_)].

Further, we performed DFT calculations to depict chemical reaction dynamics so as to understand how the stable Co_13_O_8_ cluster was formed (Fig. 3A and B), where the reaction channels for ‘O_2_ + Co_13_’ via ‘end-on’, ‘side-on’ and ‘face-on’ orientations are provided, respectively. Note that the O–O bond lengths increase significantly when an O_2_ molecule chemisorbed on the cluster surface (Fig. S15). The elongated O–O bonds of adsorptive oxygen, followed by the formation of epoxy oxygen, strengthen the subsequent reactivity of O–O bond dissociation. The following oxygen addition reactions on the Co_13_O*_n_* (*n* = 2, 4, 6) are thermodynamically favorable (Fig. 3C), with O–O bonds elongated to different degrees. We also evaluated the stability of Co_13_O_8_ by checking the likely decomposition in the presence of excessive oxygen. However, the additional oxygen molecules toward the Co_13_O_8_ cluster just absorb on the surface but do not break its cubic structure; even at 900 K, Co_13_O_8_ survives the attack of additional O_2_ molecules (Table S3). It is inferred that four oxygen molecules fully passivate a Co_13_ cluster with the eight oxygen atoms anchoring eight triangular facets toward the cubic structure.

**Figure 4.**
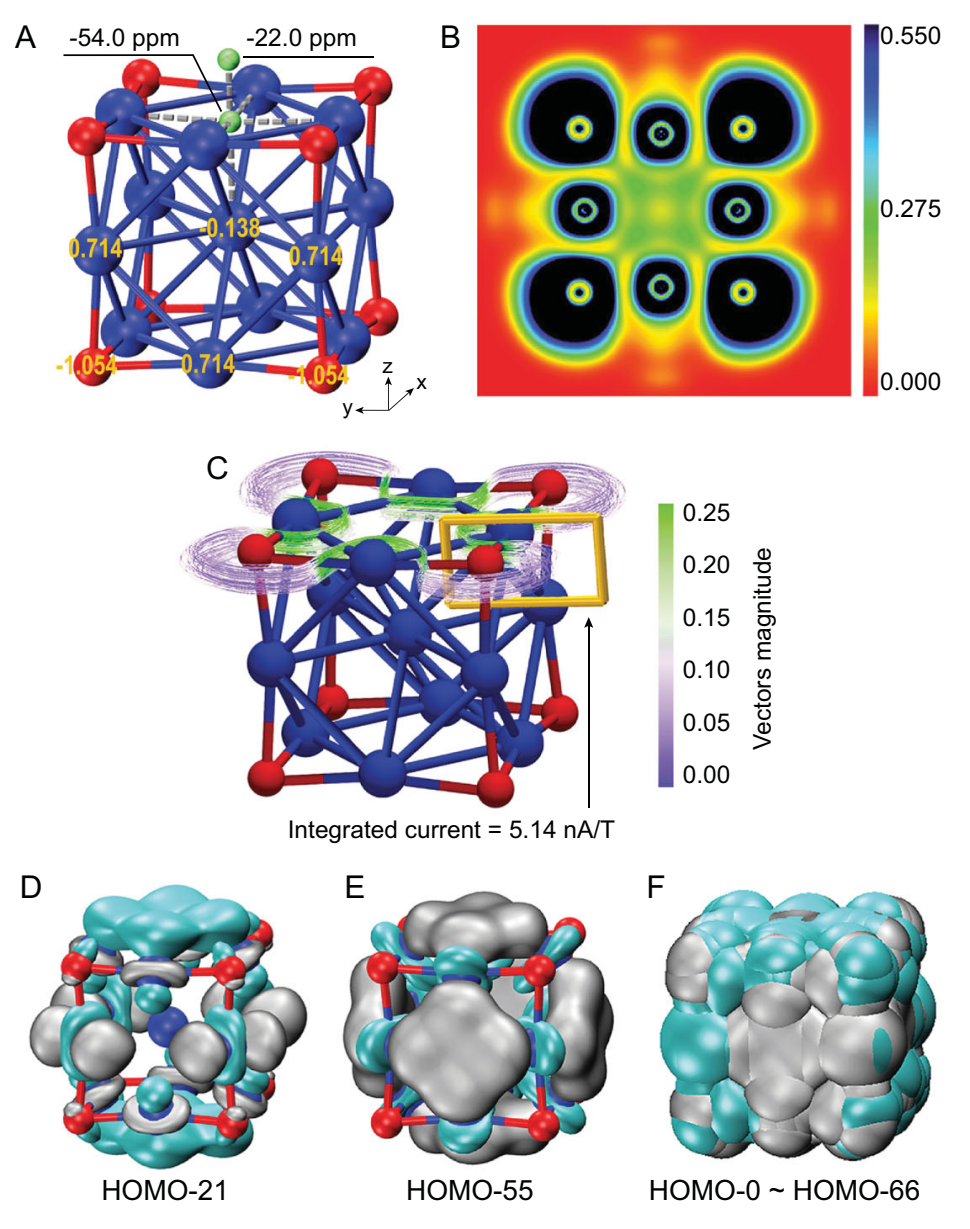
NICS, ELF, GIMIC and orbital analysis. (A) DFT calculation results of the NICS on the center of the Co_4_O_4_ plane of the cluster, with a charge of −1.054|e| on the oxygen atoms. (B) ELF analysis of charge-density distribution (e/$r_{{\rm{Bohr}}}^3$) of Co_13_O_8_ at the top-view plane. Blue and red areas represent high and low electron densities, respectively. (C) Stream tracer of the induced ring current when an external magnetic field in the [0, 0, 1] direction is applied, and the integral of the induced current that crosses the defined section is labeled. (D and E) The typical occupied orbital patterns of the Co_13_O_8_ cluster. The isosurfaces in gray and cyan refer to 0.02 and −0.02 e/Å^3^, respectively. (F) Superposition of the orbitals from HOMO to HOMO-66 showing electron delocalization with cubic aromaticity.

Further, we calculated the nucleus-independent chemical shifts (NICS) [40–43], which is often used as a criterion to evaluate the aromaticity. The NICS values were computed at a few points along the central axis of a Co_4_O_4_ plane of the cubic Co_13_O_8_. As shown in Fig. 4A, the negative NICS(0) and NICS(1) values (corresponding to the Co_4_O_4_ plane center and 1.0 Å above the plane surface) are up to −54.0 and −22.0 ppm, respectively, indicating this cluster is aromatic (larger than that of benzene; see Table S6). Meanwhile, we have also conducted electron localization function (ELF) analysis [44], which illustrates the bonding and nonbonding areas by measuring the local electron-pair density. As shown in the top-view plane (Fig. 4B), the charge-density distribution value in the central area of square with concave sides is ∼0.27e/$r_{{\rm{Bohr}}}^3$, pertaining to relatively weak metallic bond interactions between the Co atoms. In addition to covalent interactions, there is electron transfer between Co and O to form ionic bonds. Note that the electron cloud of oxygen shows obvious polarization toward the center of each plane, indicative of combined electrostatic interactions pertaining to oxygen passivation of the Co_13_ cluster.

Furthermore, we estimated the magnetically induced current density using gauge-including atomic orbitals (GIMIC) [45,46] of the Co_13_O_8_ cluster, as displayed in Fig. 4C (more details in the Supplementary data). As a result, the positive contribution of the induced current is found to be up to 5.83 nA/T, while the negative contribution is only −0.69 nA/T (i.e. an integrated net current of 5.14 nA/T with an external magnetic field perpendicular to the Co_4_O_4_ plane), suggesting remarkable aromaticity on each Co_4_O_4_ plane of the Co_13_O_8_ cluster. Figure 4D–F depicts a few typical orbitals contributing to the delocalization (more details in the Supplementary data). The multicenter delocalization of electrons accounts for its aromaticity, which, in turn, promotes cluster stability within Wade–Mingos rules analogous to polyhedral boranes, boron clusters and all-metal clusters [43,47].

**Figure 5.**
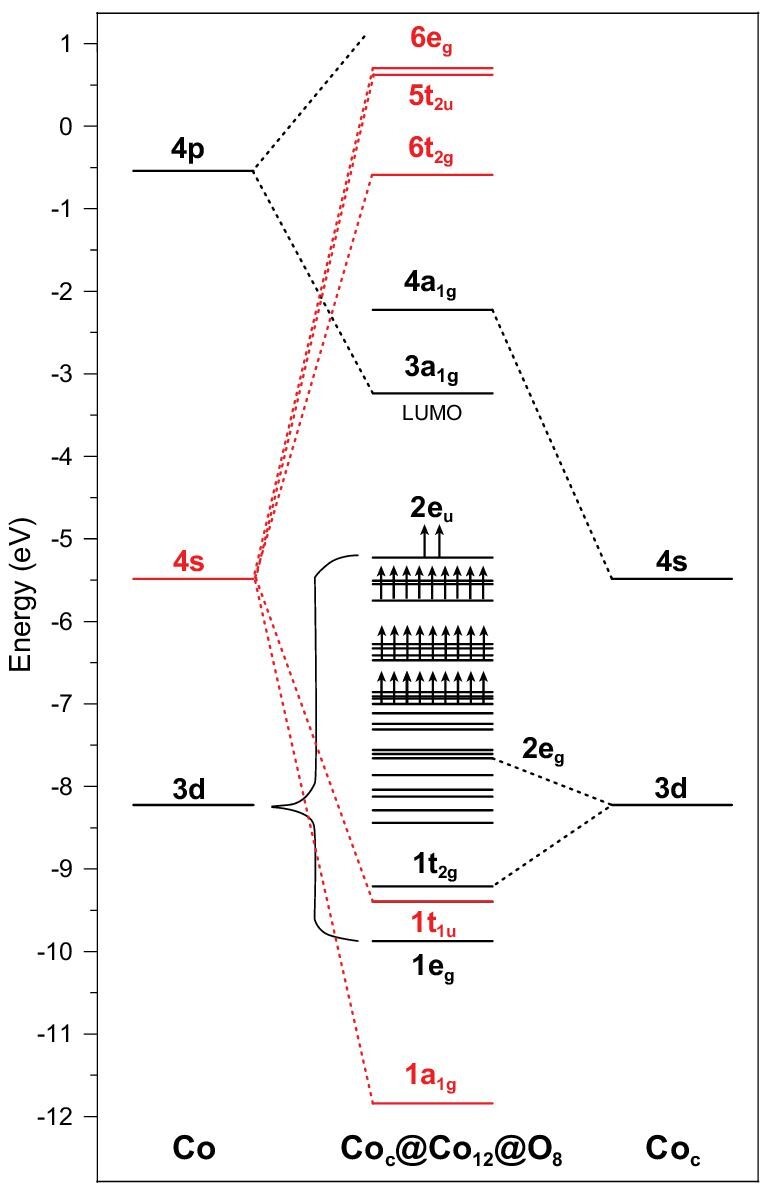
A Kohn–Sham energy-level correlation diagram of the Co@Co_12_@O_8_ cluster. The centered Co atom of Co_13_O_8_ is defined as Co_c_. The arrows are representative for spin electron occupation.

Having determined the stability, dynamics and aromaticity of the Co_13_O_8_ cluster, what is the nature of the chemical bonding and aromatic property? In order to elucidate the physical origin of the special stability of this cluster, we performed electronic structure analysis on the pseudo-cubic cluster, which can be viewed as Co@Co_12_@O_8_ for the sake of bonding analysis. Figure 5 depicts the Kohn–Sham energy levels from interactions among Co_12_, O_8_ and the central Co atom (labeled as Co_c_). The Co 3d-orbitals span a narrow 3d-band with 65 orbitals because of the relatively small orbital overlap between the neighboring Co atoms connected with a Co–Co distance of ∼2.4 Å. This narrow 3d-band allows hosting a large number of magnetically coupled, unpaired electrons on each Co center. In contrast, the radially more diffused Co 4s-orbitals form a much larger manifold of group orbitals of symmetry a_1g_, t_1u_, t_2g_, e_g_ and t_2u_, respectively. Among them, the a_1g_ and t_1u_ orbitals are strongly bonding for Co_12_ cage, thus lying in the low-energy end of the 3d-band. The weakly antibonding orbitals t_2g_ and e_g_ of the Co_12_ cage are destabilized by the Co_c_ atom (with 3d-orbitals in t_2g_*_ _+ *e_g_ symmetry) so much that they become strongly antibonding orbitals in Co@Co_12_. As a result, the Co_12_ cage can only hold eight electrons in the 4s-based a_1g_ and t_1u_ orbitals to form an (a_1g_)^2^(t_1u_)^6^ configuration. This scenario is reminiscent to [Zn^(I)^_8_] and [Mn^(I)^_8_] clusters within a ‘6n + 2’ rule of electron counting and having so-called cubic aromaticity [48,49].

As a metal oxide cluster, Co_13_O_8_ possesses special stability due to the cubic aromaticity from multicenter Co–Co metal–metal bonding. The electronic structure analysis shows that the oxidation states of the atoms in this cluster can be formally assigned as central Co^(0)^, cage Co^(^^4/3^^+^^)^, and O^(^^2^^−^^)^, respectively, which are consistent with their calculated net charges and spin distribution (Table S7). Here, the Co_12_ cage (in cubic Co_13_O_8_ cluster) with 12 quasi-monovalent Co (I, 3d^7^4s^1^) atoms is forced to lose 4 extra electrons because of the antibonding interaction with the 3d-orbitals of the central Co atom (Fig. 5). The calculated spin density populations support this chemical bonding analysis (for details see Table S7). Also, we searched for the lowest energy spin states of Co@Co_12_O_8_, and found that a total spin quantum number *S *= 29/2 corresponds to the lowest energy state. On this basis, we calculated the total magnetic moment of Co_13_O_8_ being up to 30*μ*_B_, simply by using ${\mu _{\rm{s}}} = \ g\sqrt {S( {S + 1} )} {\mu _{\rm{B}}}$ (the Landé factor *g* = 2.0023), which is larger than the usual cobalt oxides [50].

## CONCLUSION

In summary, utilizing self-developed DUV-LIMS technique that takes advantage of high-efficiency photoionization of neutral cobalt clusters, here we observe the reactions of cobalt clusters with oxygen and discover the prominent stability of Co_13_O_8_. Theoretical calculations based on different methods concur with this experimental finding, and unveil its distinctive stability pertaining to a perovskite-like body-centered cubic structure. Thermodynamics and reaction dynamics involving structural evolution from icosahedron Co_13_ to the cubic Co_13_O_8_ are addressed. We name this kind of clusters as ‘metalloxocubes’ to stimulate further research interest in exploring such materials with well-defined components and regular structures. This class of neutral oxygen-passivated metal clusters is a reasonable candidate for genetic materials in view of the cubic nature of the building blocks and the special stability from cubic aromaticity.

## METHODS

### Experimental

The experiments were carried out utilizing a customized reflection time-of-flight mass spectrometer (Re-TOFMS) combined with the newly developed 177.3-nm deep-ultraviolet laser (details in the Supplementary data) [51]. The optimized Re-TOFMS, LaVa source and deep-ultraviolet laser ensure highly efficient preparation and detection of well-resolved neutral cobalt clusters under a normal distribution. Following the generation of cobalt clusters, a tangential deflection electric field (DC 200 V) was designed to remove any charged particles to attain neutral clusters. The homemade LaVa source is coupled with a reaction cell downstream (6 mm diameter, 6 cm long), allowing for sufficient collision reactions (∼30 Pa pressure) with varied reactants (e.g. 3–20% O_2_ seeded in He), controlled by a pulsed general valve with the on-time duration to be set as 150–250 μs per period of 100 ms (i.e. a frequency of 10 Hz). The neutral cluster beam and reaction products were then collimated into another high-vacuum TOF chamber through a skimmer (*φ* 2 mm). At the arrival in the ionization zone (i.e. the space between the first and second acceleration plates), the cluster beam meets the deep-ultraviolet laser from the coaxial front direction; simultaneously, the acceleration voltages are triggered so that the instantaneously ionized neutral clusters are analyzed by the Re-TOFMS.

### Theoretical methods

Three methods are used to search and identify the global-minimum structures of the clusters in this study. The first one is CALYPSO approach based on the particle swarm optimization method [52]. Also, here we have used a homemade code strategy (see Appendix in the Supplementary data) based on the graph theory method [53] to help find the ground-state structures by taking into consideration the prototypes of crystal structures of metal cobalt, rock salt and spinel cobalt oxides. This is based on chemical knowledge that metal clusters tend to a closest stacking mode. This approach has equivalent efficiency to find energy-minimum structure of small clusters (e.g. *n* < 10) as nascent CALYPSO code, but shows faster speed for larger clusters (Fig. S9). Following DFT calculations of energetics were performed via a PWmat software package [54,55] in a plane-wave pseudopotential basis set, with a 20 Å vacuum space set in the *x*, *y* and *z* directions. Spin polarization was considered in all the calculations. Perdew–Burke–Ernzerhof exchange correlation functional [56] with SG15 pseudopotential and DFT-D2 van der Waals corrections were applied in the calculations. A Hubbard-*U* model based on PWmat code was employed to correct the strong-correlation Coulomb interaction between d-orbital electrons on Co atoms.

We also conducted independent basin-hopping global-minimum search using TGMin code to conclusively determine the cluster structure of Co_13_O_8_. Also, DFT calculations were carried out with Gaussian-16 quantum chemical package [57]. Using the standard 6-311G basis set augmented by 3df polarization, the molecular orbitals and ELF patterns are calculated by combining Becke's exchange and Perdew–Wang's correlation functionals (denoted BPW91). The program package of Multiwfn [58] is utilized to analyze the ELF and orbitals. Further sophisticated calculations of electronic structure and NICS [40–43] were performed via ADF code [59].

## Supplementary Material

nwaa201_Supplemental_Files
