# Supplementary material for: Co13O8—metalloxocubes: a new class of perovskite-like neutral clusters with cubic aromaticity
Source: Natl Sci Rev. 2020 Aug 29;8(1):nwaa201. doi: 10.1093/nsr/nwaa201 (PMC8528261; doi:10.1093/nsr/nwaa201)
Supplement: nwaa201_Supplemental_Files [file nwaa201_Supplemental_Files.zip › NSR-ESI__Co13O8.docx]

**Supplementary Materials for**

**Co_13_O_8_—Metalloxocubes: A New Class of Perovskite-like Neutral Clusters with Cubic Aromaticity**

Lijun Geng^1,3^, Mouyi Weng^2^, Cong-Qiao Xu,^4^ Hanyu Zhang^1^, Chaonan Cui^1^, Haiming Wu^1^, Xin Chen^2^, Mingyu Hu^2^, Hai Lin^2^, Zhen-Dong Sun^3^, Xi Wang^5^, Han-Shi Hu^6^, Jun Li^4,6^, Jiaxin Zheng^2^, Zhixun Luo^1^*, Feng Pan^2^* & Jiannian Yao^1,2^*

^1^Beijing National Laboratory for Molecular Sciences (BNLMS), State Key Laboratory for Structural Chemistry of Unstable and Stable Species, Laboratory of Photochemistry, Institute of Chemistry, Chinese Academy of Sciences, Beijing 100190; University of Chinese Academy of Sciences, Beijing 100049, China.

^2^School of Advanced Materials, Peking University Shenzhen Graduate School, Shenzhen 518055, China.

^3^School of Physics, Shandong University, Jinan 250100; School of Physics and Electrical Engineering, Kashi University, Kashgar 844006, China.

^4^Department of Chemistry, Southern University of Science and Technology, Shenzhen 518055, China.

^5^College of Science, Beijing Jiaotong University, Beijing 100044, China.

^6^Department of Chemistry and Key Laboratory of Organic Optoelectronics and Molecular Engineering of Ministry of Education, Tsinghua University, Beijing 100084, China.

*Corresponding authors. Emails: [zxluo@iccas.ac.cn](mailto:zxluo@iccas.ac.cn); [panfeng@pkusz.edu.cn](mailto:panfeng@pkusz.edu.cn); [jnyao@iccas.ac.cn](mailto:jnyao@iccas.ac.cn)

**Contents**


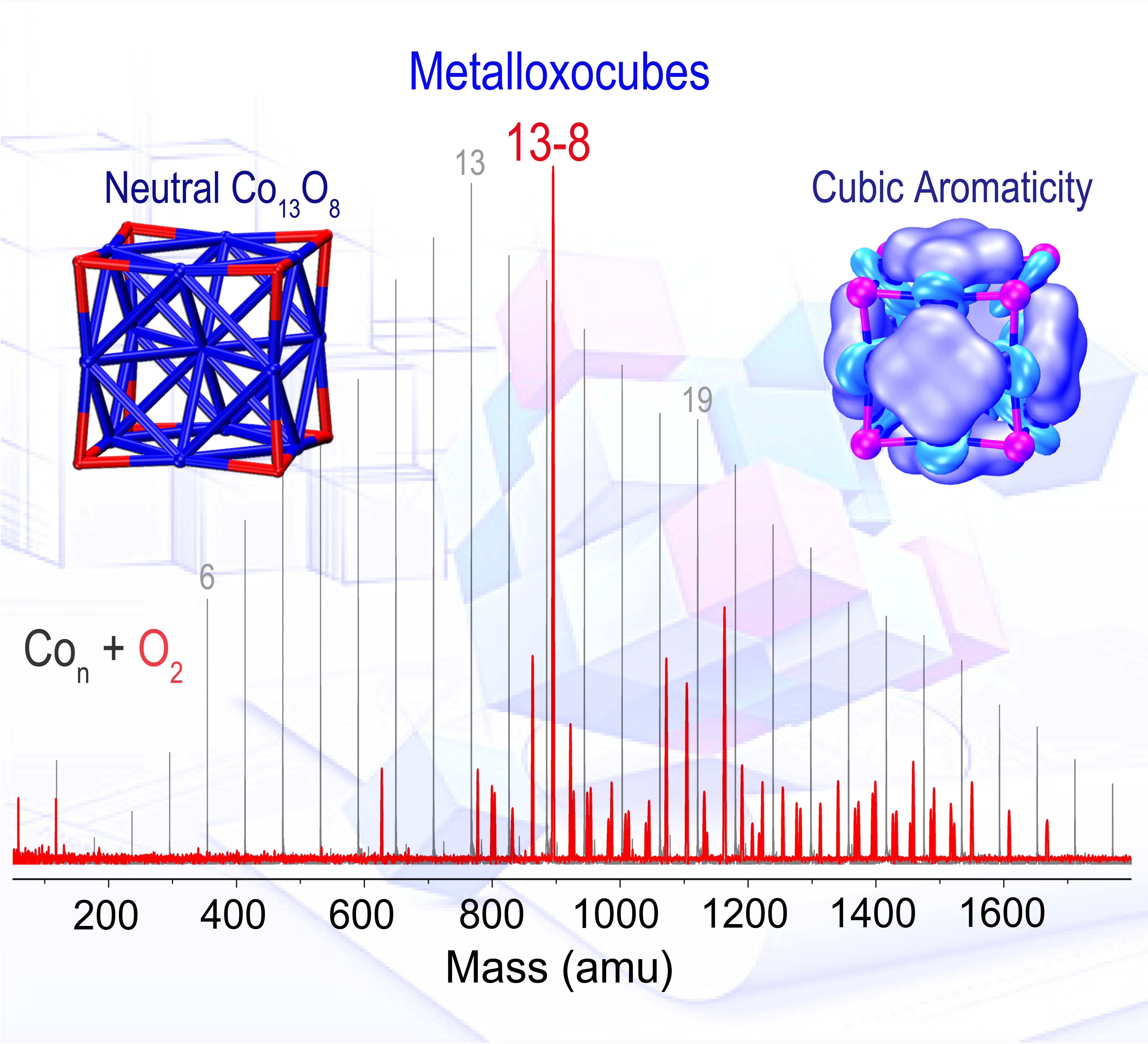


[Section 1. Experimental details 3](#_Toc44143493)

[Section 1.1 Experimental methods 3](#_Toc44143494)

[Section 1.2 Details of Co_n_ clusters reacting with oxygen 5](#_Toc44143495)

[Section 1.3 Repeated experiments 8](#_Toc44143496)

[Section 2. Extended experiments on Co_n_^±^, Fe_n_^+^ and Ni_n_^+^ 9](#_Toc44143497)

[Section 3. Theoretical and computational details 11](#_Toc44143498)

[Section 3.1 Structure search and optimization 11](#_Toc44143499)

[Section 3.2 Energy calculation and thermodynamics 15](#_Toc44143500)

[Section 3.3 Spin multiplicity 16](#_Toc44143501)

[Section 3.4 Dynamic structure transition in physical networks 17](#_Toc44143502)

[Section 3.5 Reaction dynamics calculations 18](#_Toc44143503)

[Section 3.6 NICS and spin density population 21](#_Toc44143504)

[Section 3.7 The bond lengths and natural population analysis 23](#_Toc44143505)

[Section 3.8 Molecular orbital analysis 24](#_Toc44143506)

[Section 3.9 Ionization energies of Co_n_O_m_ clusters 25](#_Toc44143507)

[Section 3.10 Co_13_O_8_ ring current 28](#_Toc44143508)

[References 30](#_Toc44143509)

# Section 1. Experimental details

## Section 1.1 Experimental methods

The complete experimental device (Supplementary Fig. 1) consists of four components, i.e., the customized vacuum system, the laser evaporation (LaVa)[1-9] system for efficient cluster generation in tandem with a flow tube reactor[10-20], and a home-made reflection time-of-flight mass spectrometer (Re-TOFMS) which is combined with the newly-developed deep-ultraviolet ionization laser (177.3 nm wave length, 15.5 ps pulse width, 10 Hz repetition rate, ~15 μJ pulse energy used in this study) [21-27]. When running laser ablation experiments, typically a rotating cobalt metal disk (99.95%) at the focused radiation, a 532 nm laser (Nd: YAG) with a pulse energy of ~35 mJ and a repetition rate of 10 Hz was used in the study. We used a pulsed valve (Parker, Serial 9) from the rear for He buffer gas inlet (10.0 atm pressure, 99.999% purity, ∼230 µs duration) and a stainless-steel nozzle (ɸ= 2 mm, L= 35 mm) at the outlet head, enabling the production of well-resolved cobalt clusters within supersonic expansion.

Prior to introducing reactants of vary quantities, a pre-test was conducted by evaluating the range of minimum and maximum pulse widths of the buffer gas helium and the proper concentrations of the reactant oxygen, so as to exclude the influence of thermal radiation on hot cluster decay or shift of size distribution in the successive collection of experimental data [28]. Considering that the molecule density (ρ) is proportional to the molecule number (N) of per pulsed reaction gas and inversely proportional to the gas flow time (t), velocity (ν) and cross-section area (S) of the reaction tube, that is, ρ=N/(t∙S∙ν), the reactant molecule density in the flow tube reactor is estimated to be approximately 10^19^~10^20^ molecules per cubic meter when the on-time condition of the pulsed valve (10 Hz) was set to be within a range of 100-250 μs. Downstream the cluster beam, a tangential deflection voltage (DC 200 V) was employed to remove charged ions. Subsequently, the neutral cluster beam was collimated into the TOF chamber via a ɸ 2 mm skimmer. At the arrival of neutral clusters into the ionization zone (i.e., the space between the first and second electrode plates), they meet the ionization laser from a coaxial front direction. After ionization by the deep-ultraviolet laser, the neutral clusters were then analyzed by the Re-TOFMS. Considering that the ionization energies of most metal atoms are centered at 7 eV, this Re-TOMFS instrument combined with the 177.3 nm deep-ultraviolet (DUV)[24, 25] laser bears unique capability for single photon ionization[22, 29-32] of neutral metal clusters.


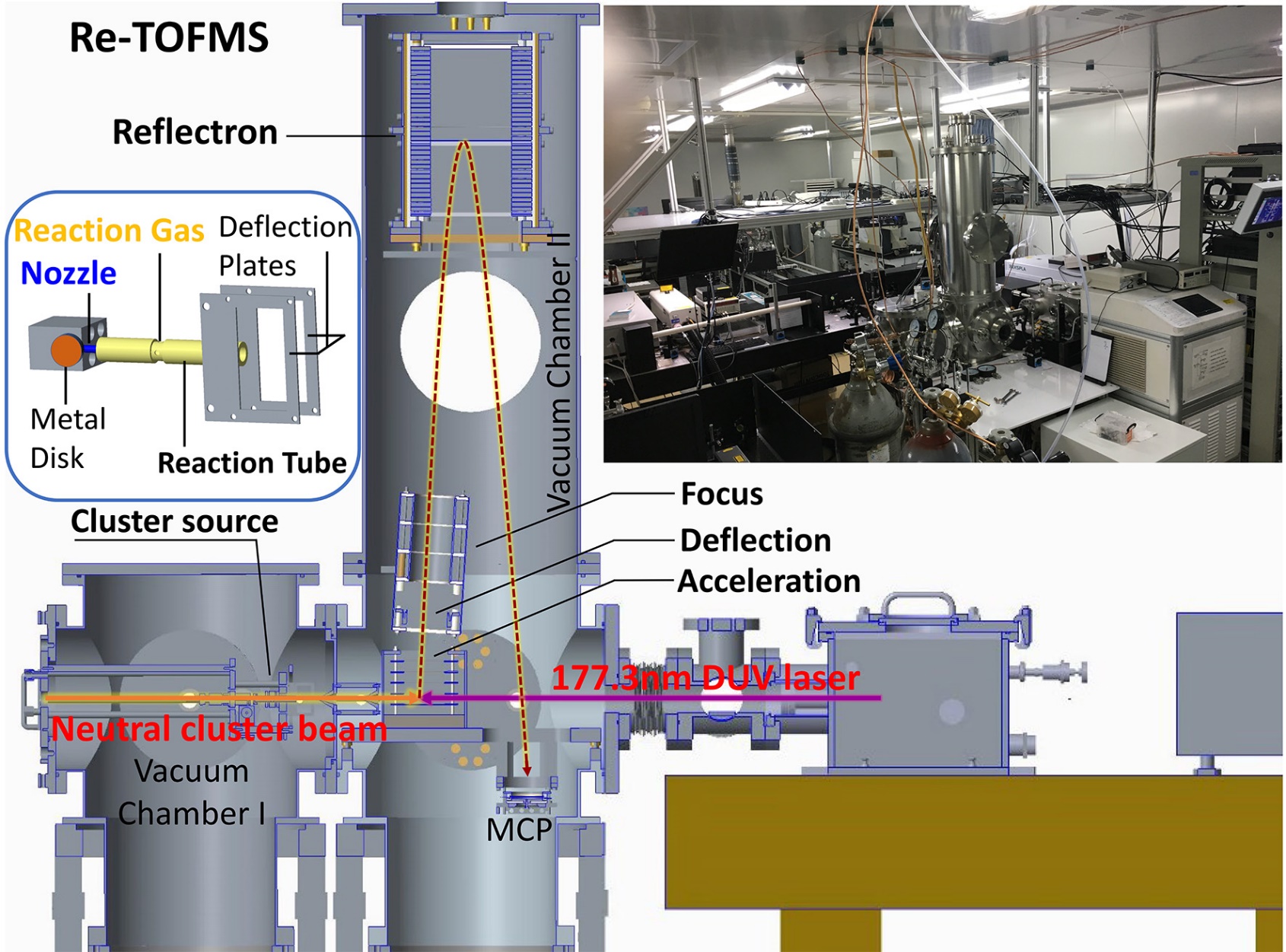


**Supplementary Figure 1.** Experimental instrumentation. A sketch showing the home-made reflection time-of-flight mass spectrometer (Re-TOFMS), combined with the customized deep ultraviolet (DUV) 177.3 nm ps-pulsed laser for photoionization.

## Section 1.2 Details of Co_n_ clusters reacting with oxygen

Supplementary Fig. 2 displays the mass spectra at the absence and presence of slowly increased amounts of oxygen. It is notable that, even with trace amount of oxygen being contaminated, the cobalt clusters readily form O_2_-adsorbed species which displays increased mass abundances with more oxygen being introduced. From trace amount oxygen to a large gas flow, the Co_13_O_8_ consistently keep growing. The stability of Co_13_O_8_ is associated with its body-centered cubic structure analogous to perovskite.


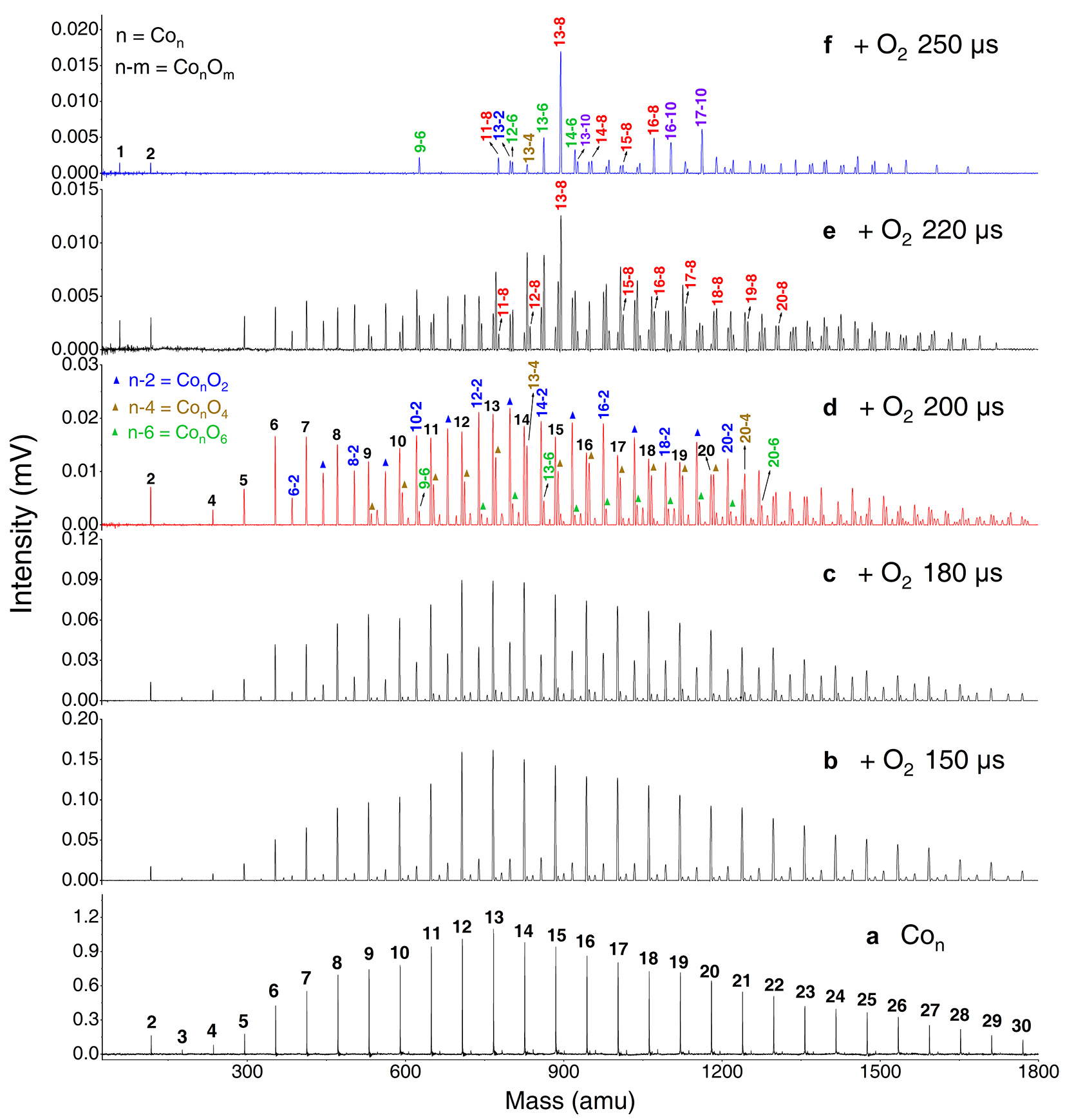


**Supplementary Figure 2.** Experiments of varied oxygen. **a**, Experimental details showing the size distribution of the nascent Co_n_ clusters. **b-f,** The mass spectra of Co_n_ clusters after reacting with different amounts of oxygen (1.0 atm 20% O_2_/He) being introduced into the flow tube, controlled by a pulsed valve with varying pulse widths at 150 µs, 180 µs, 200 µs, 220 µs, 250 µs respectively.

Supplementary Fig. 3 shows the three-dimensional histograms of the integral signal intensities of the observed Co_n_O_m_ clusters corresponding to curve d in Supplementary Fig. 2 (also Fig. 1B in the main text). It is conspicuous that Co_13_O_2_, Co_13_O_4_, and Co_13_O_6_ are dominant in the series of Co_n_O_2_, Co_n_O_4_, and Co_n_O_6_, respectively.


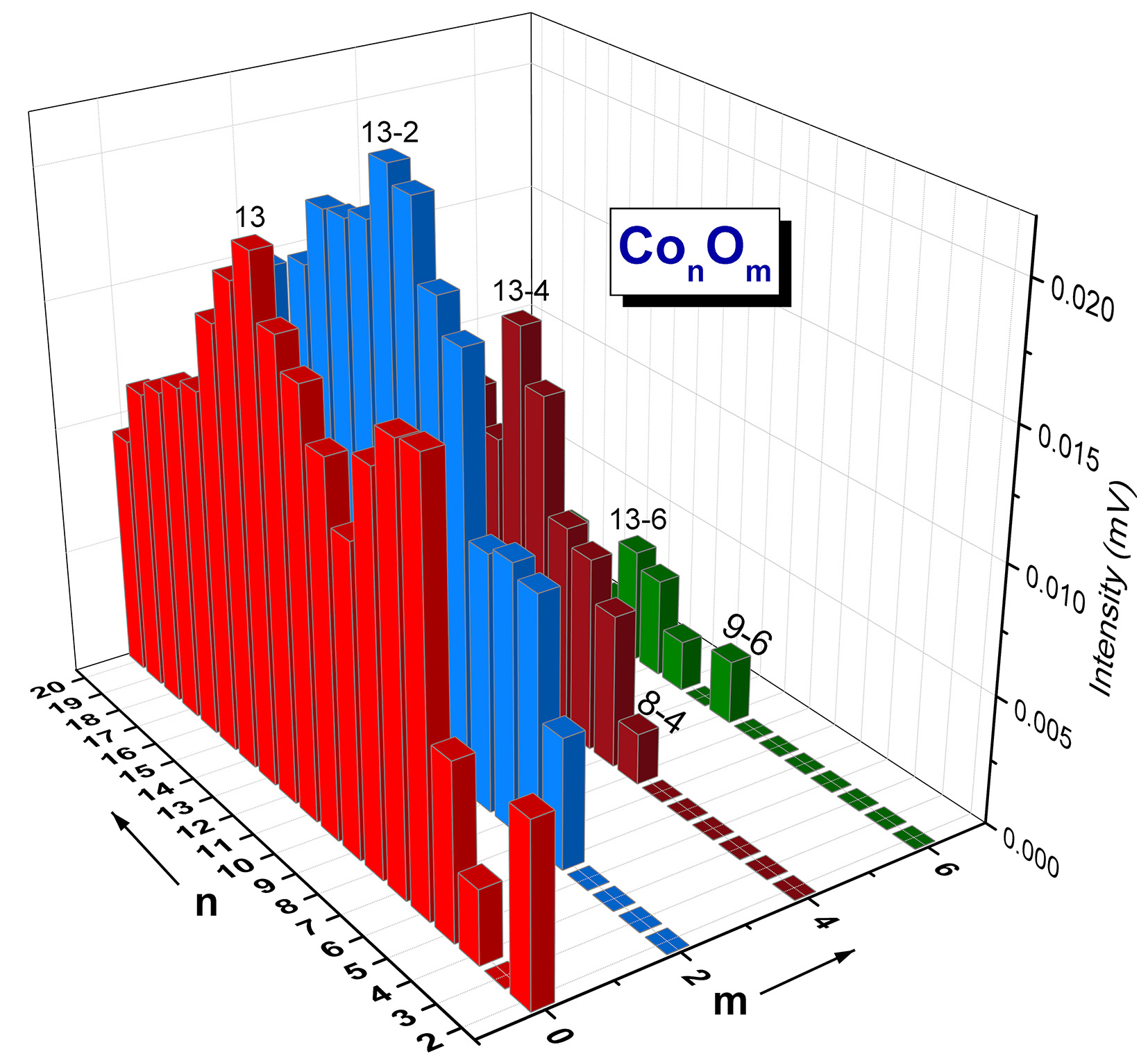


**Supplementary Figure 3.** Histogram of mass abundances. Three-dimensional histograms of the integral signal intensities of the observed typical Co_n_O_m_ clusters corresponding to curve d in Supplementary Fig. 2. The Co_n_ and Co_n_O_m_ clusters are ionized by the ps-pulsed deep-ultraviolet 177.3 nm laser.

Supplementary Fig. 4 plots the integral intensities of the mass peaks for typical Co_n_ and Co_n_O_m_ clusters (n=12-14, m=2,4,6,8) shown in Supplementary Fig. 2. In the presence of small amount of oxygen, the Co_n_O_2,4_ series emerge first, followed by the formation of Co_n_O_6,8_. When a large amount of oxygen is introduced to the flow reactor, Co_13_O_8_ displays constantly increased mass abundances.


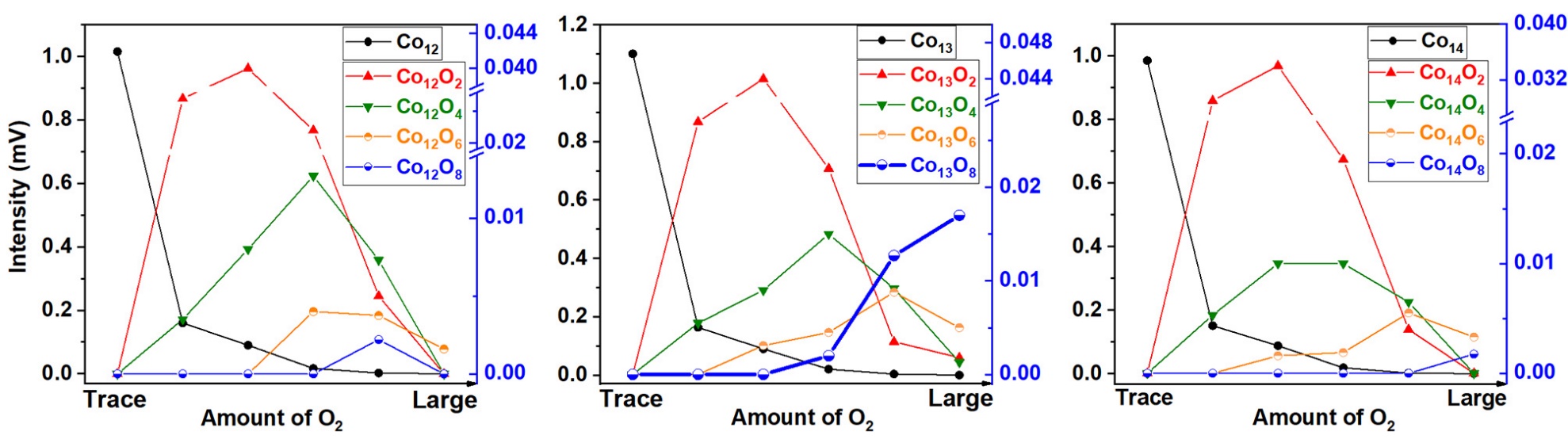


**Supplementary Figure 4.** Variation of mass abundances. The varied intensities of neutral Co_n_ and Co_n_O_m_ (n=12-14, m=2,4,6,8) change with the amount of reactant gas O_2_, from trace to large, controlled by the pulse valve, with varied pulse width at 150 µs, 180 µs, 200 µs, 220 µs, 250 µs respectively.

## Section 1.3 Repeated experiments

We tried experiments with different oxygen concentrations (e.g., 10% O_2_/He, 40% O_2_/He, etc.), and found the same results that Co_13_O_8_ is the only dominant species for neutral cobalt clusters in reacting with large oxygen. Supplementary Fig. 5 presents another set of experiments where the mass distribution centered at Co_12_, however, Co_13_O_8_ still survive the reactions with oxygen as the final dominant product. Furthermore, the original size distribution of Co_n_ clusters is well recovered after stopping the introduction of oxygen reactant.


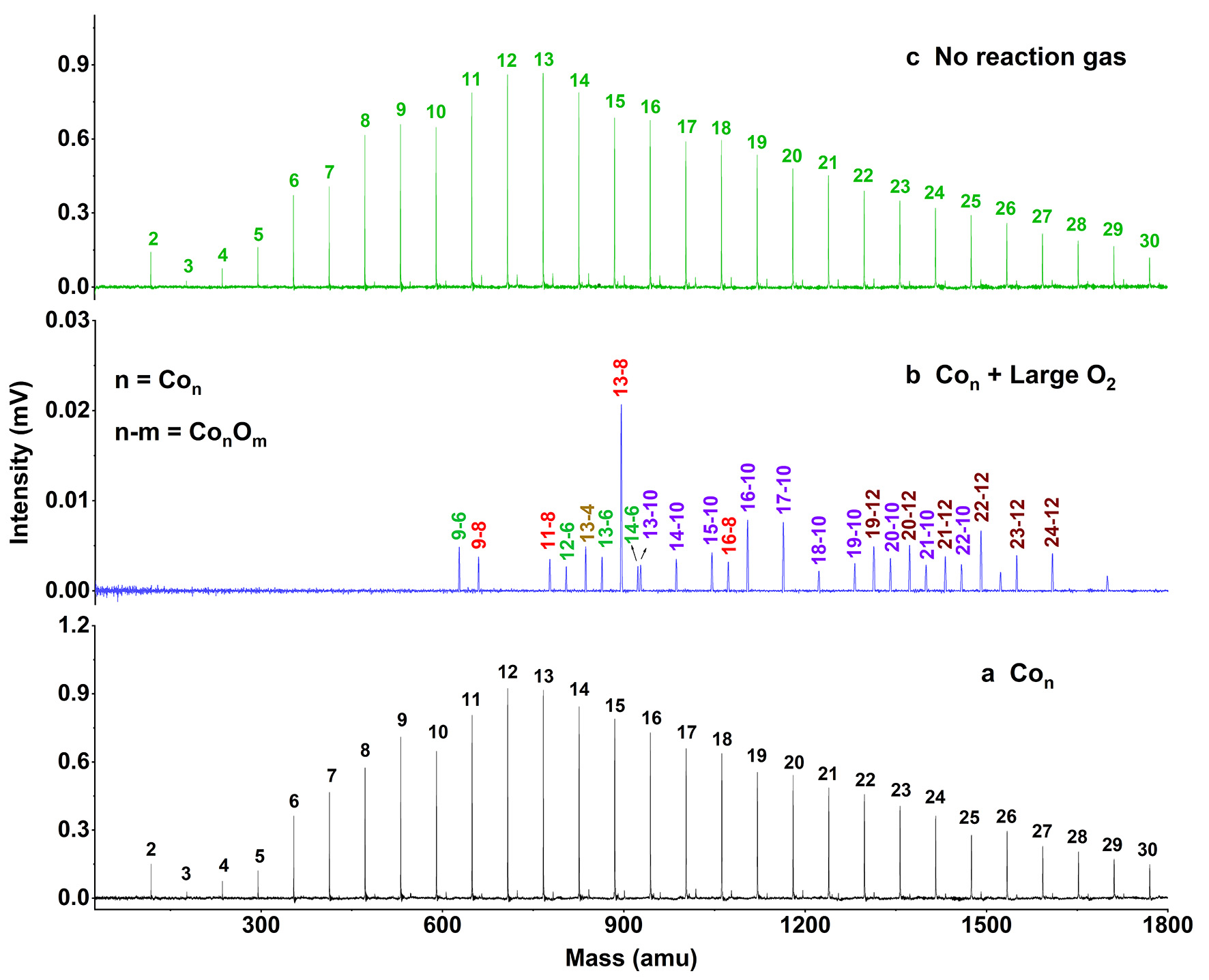


**Supplementary Figure 5.** Repeated experiments. **a,** A repeated experiment with altered representative size distribution of the nascent Co_n_ clusters. **b,** The mass abundance after react with 1.0 atm 40% O_2_/He introduced in the mini flow tube by the pulse width of the pulse valve in 250 µs. **c,** The mass spectrum after the stop of introducing oxygen reactant. The Co_n_ and Co_n_O_m_ clusters are ionized by the 177.3 nm deep-ultraviolet laser.

# Section 2. Extended experiments on Co_n_^±^, Fe_n_^+^ and Ni_n_^+^

Besides the cobalt cluster neutrals, we also observed the reactions of cationic and anionic cobalt clusters with oxygen. A clean mass distribution corresponding to Co_1-33_^+^ is shown in Supplementary Fig. 6a, where a decreasing tendency exists from n=1-3 and the other Co_n_^+^ clusters display a Gaussian distribution centered at Co_11_^+^. In the presence of oxygen, a varied of Co_n_O_m_^+^ products emerge in the mass spectra, with Co_n_O_4_^+^ and Co_n_O_6_^+^ series displaying reasonable mass abundances. In particular, the Co_13_O_8_^+^ becomes dominant in the mass spectra in the presence of large flow rate of oxygen. This is consistent with the finding on neutrals as shown above. Also, we have managed to observe the reactions of anionic cobalt clusters Co_n_^–^ (n≤33) with oxygen, and found there is similar case (Supplementary Fig. 6b). When anionic cobalt clusters react with a large amount of oxygen, Co_13_O_8_^–^ also stands out showing the highest mass abundance among all the observed cobalt clusters and their oxides.


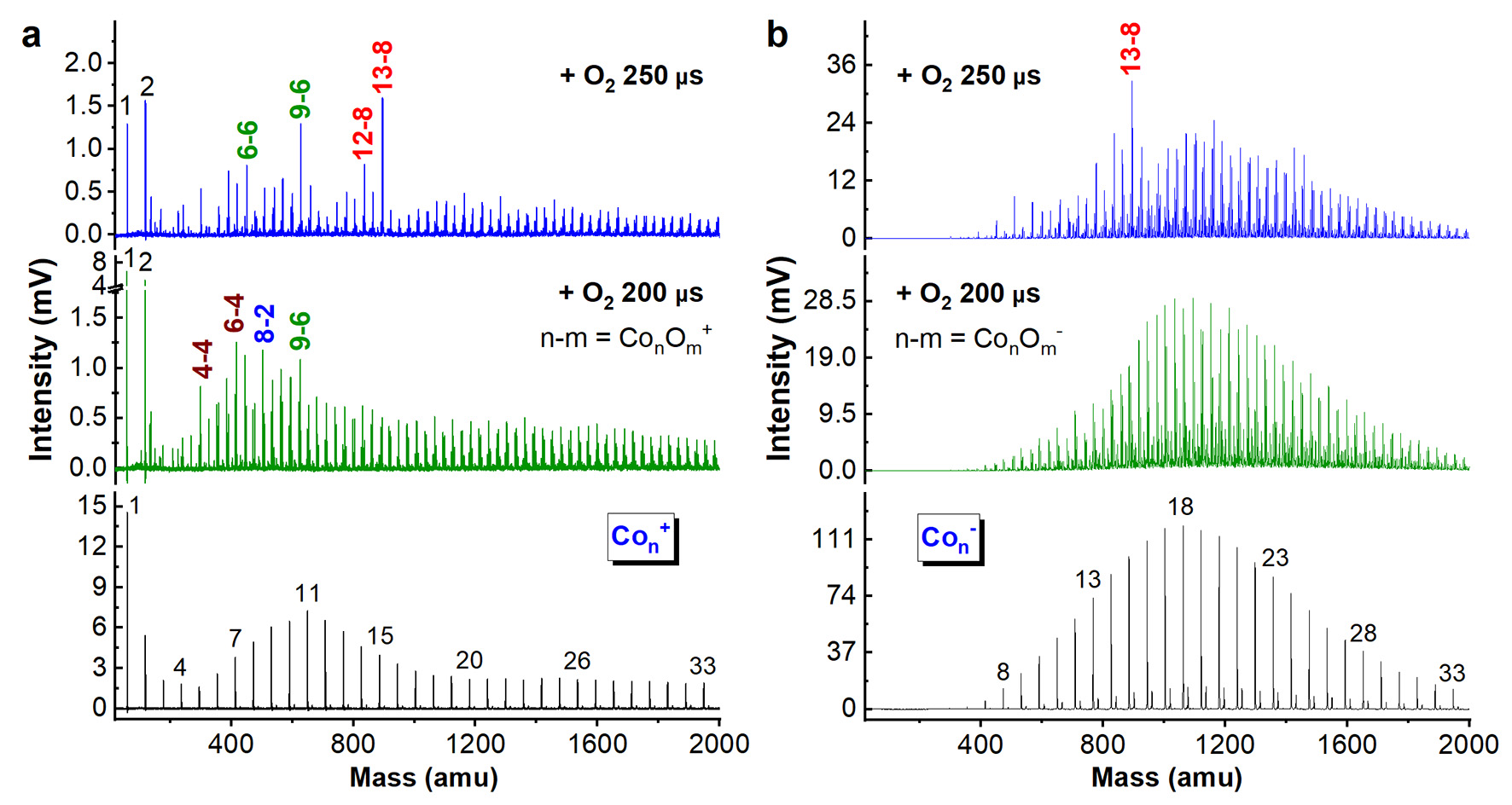


**Supplementary Figure 6.** Extended experiments on cationic Co_n_^+^ and anionic Co_n_^–^ clusters. **a/b,** TOF mass spectrometry of cationic and anionic cobalt clusters formed in helium buffer gas, after reacting with 1.0 atm 5% O_2_/He introduced in the mini flow tube, with altered pulse widths at 200 µs and 250 µs respectively. The anions were directly detected by adding similar negative voltages to the electrode plates of accelerated field.

Similarly, we have also tried iron and nickel systems. While it seems more challenging to obtain well-resolved clean mass distribution of neutral Fe_n_ and Ni_n_ clusters of similar size distribution, we have managed to observe the cationic iron and nickel clusters and monitored the reactions of Fe_n_^+^ and Ni_n_^+^ with oxygen. As results, similar results are found pertaining to unique stability of such a class of M_13_O_8_ clusters, as shown in Supplementary Fig. 7 and Fig. 8.


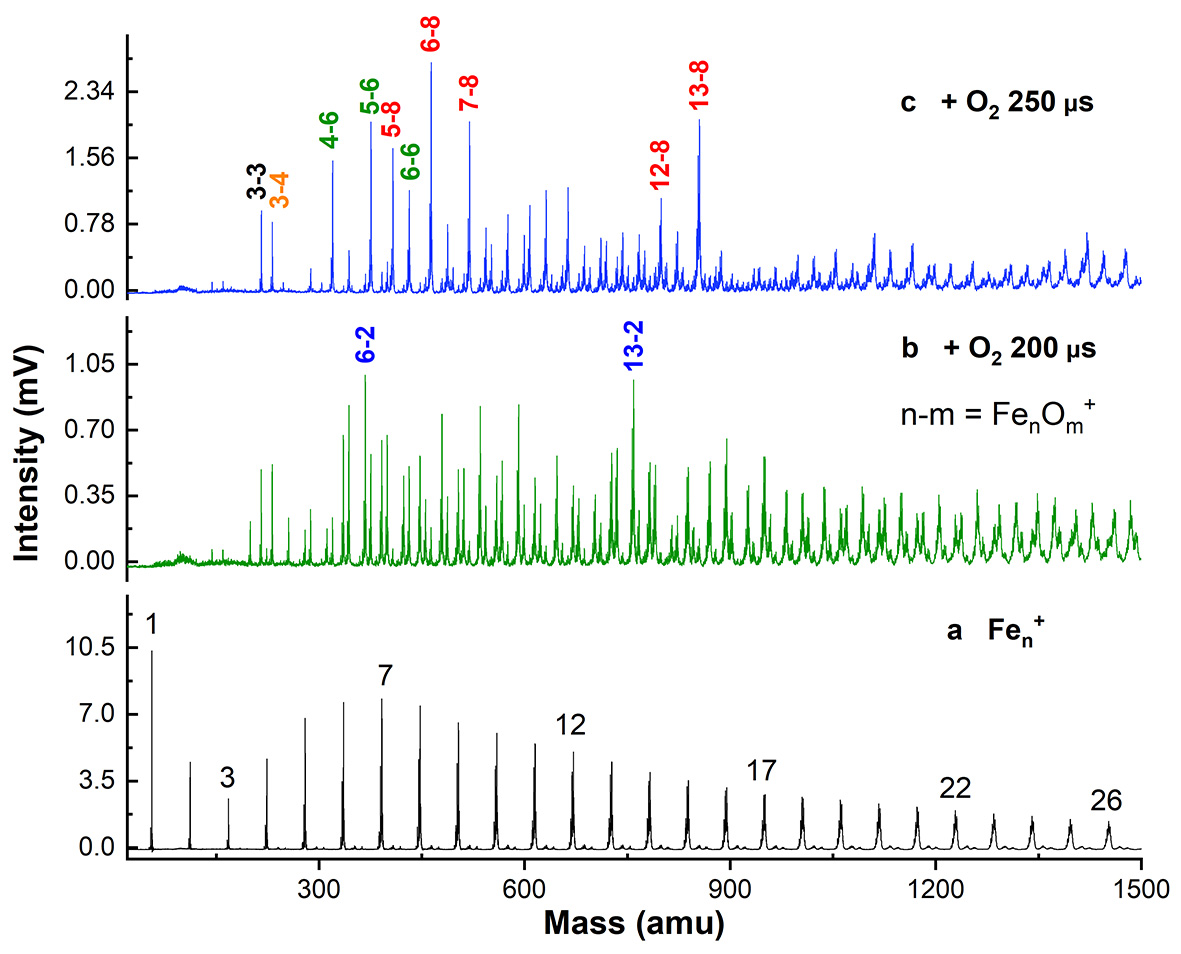


**Supplementary Figure 7.** Extended experiments on cationic Fe_n_^+^ clusters. TOF mass spectrometry of cationic Fe_n_^+^ clusters formed in helium buffer gas, after reacting with 1.0 atm 5% O_2_/He introduced in the mini flow tube, with altered pulse widths at 200 µs and 250 µs respectively.


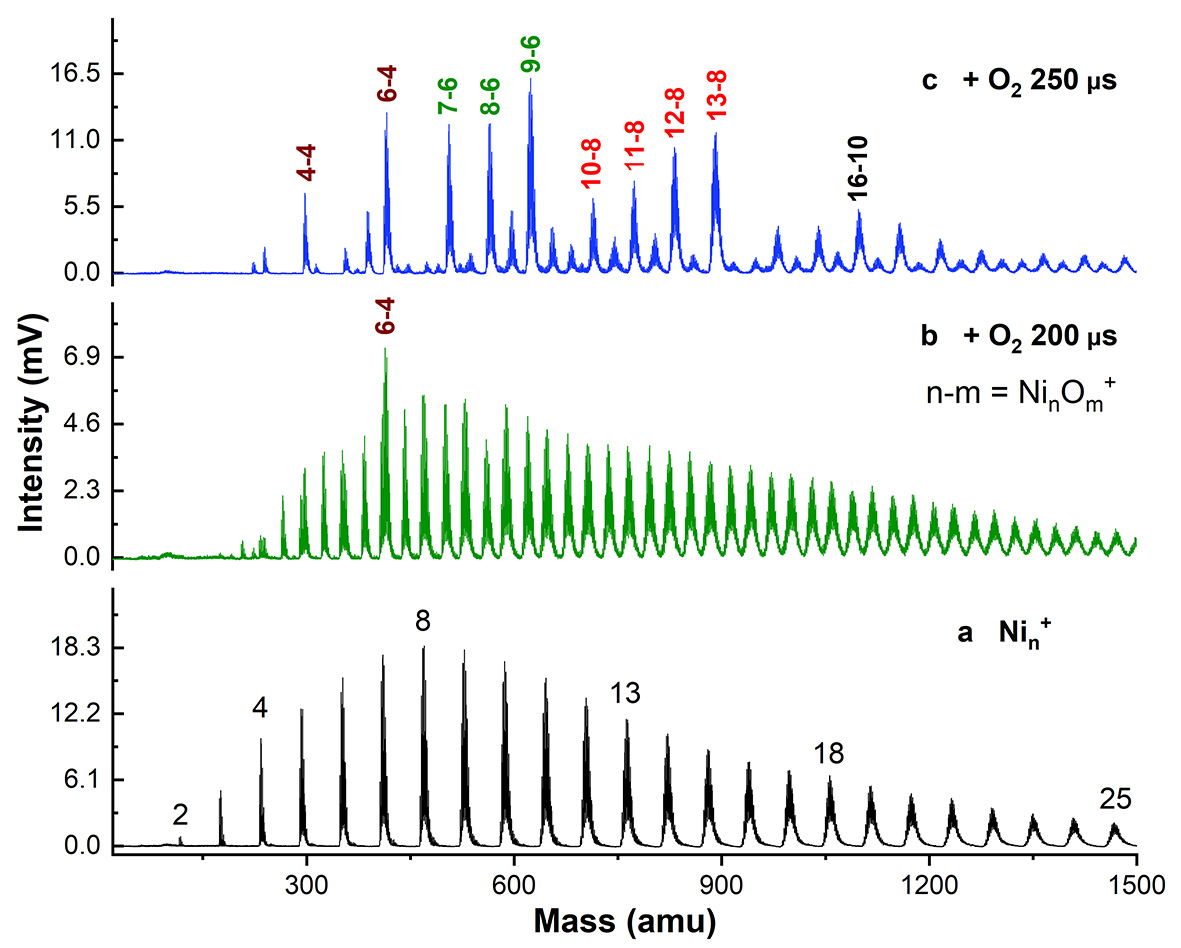


**Supplementary Figure 8.** Extended experiments on cationic Ni_n_^+^ clusters. TOF mass spectrometry of cationic Ni_n_^+^ clusters formed in helium buffer gas, after reacting with 1.0 atm 5% O_2_/He introduced in the mini flow tube, with altered pulse widths at 200 µs and 250 µs respectively.

# Section 3. Theoretical and computational details

## Section 3.1 Structure search and optimization

*3.1.1 Structural search code CALYPSO & pre-search procedure*

Three methods were used to search and identify the global minimum structures of Co_n_O_m_ clusters. Two of them are based on CALYPSO code. The first one is the general strategy of CALYPSO code based on the particle swarm optimization method[33-39]. During CALYPSO code searching, a generation of 10 species was used.

For the larger oxide clusters, we introduced a pre-search procedure to find the closest stacking clusters in Co-O crystal structures prototype to accelerate the searching process. This is based on the general chemical knowledge that metal clusters tend to build a structure within the most stacking mode. As shown by a comparison in Supplementary Figure 9, this pre-search method has equivalent efficiency to find energy-minima structure of small clusters (e.g., n<10) as nascent CALYPSO code, but it shows faster speed for larger clusters. As the number of atoms increases, atoms tend to arrange in the most stacking mode as that in bulk system, the closest stacking structures strategy tends to have more advantages for the larger ones.

The details to find the most stacking clusters from a crystal structure prototype is given as below. For a Co_n_O_m_ cluster, we need to choose n cobalt atoms and m oxygen atoms from a crystal supercell, which has the minimum total distance as described below.

$d_{\min}=\left\{ \sum_{i,j;i\neq j} \left| r_{i,p}-r_{j,p} \right|^{2} \right\}_{\min}$ (s1)

Here i and j are atomic index ranging from 1 to the total number of atoms in the cluster; r_i,p_ and r_j,p_ are the atomic coordinates of the atoms (number i, j) in the cluster (number p) respectively; while d_min_ refers to the minimum total distance among all the possible clusters. A graph theory code to find the nearest neighbor was used to help us to design a depth first searching (DFS) algorithm for the most stacking mode structure. Atoms of inner layer is considered first as they could have more possibility to build a cluster with less total distance. The script code to find a most stacking mode cluster from crystal structures can be found in a compressed package in Appendix for free to share.


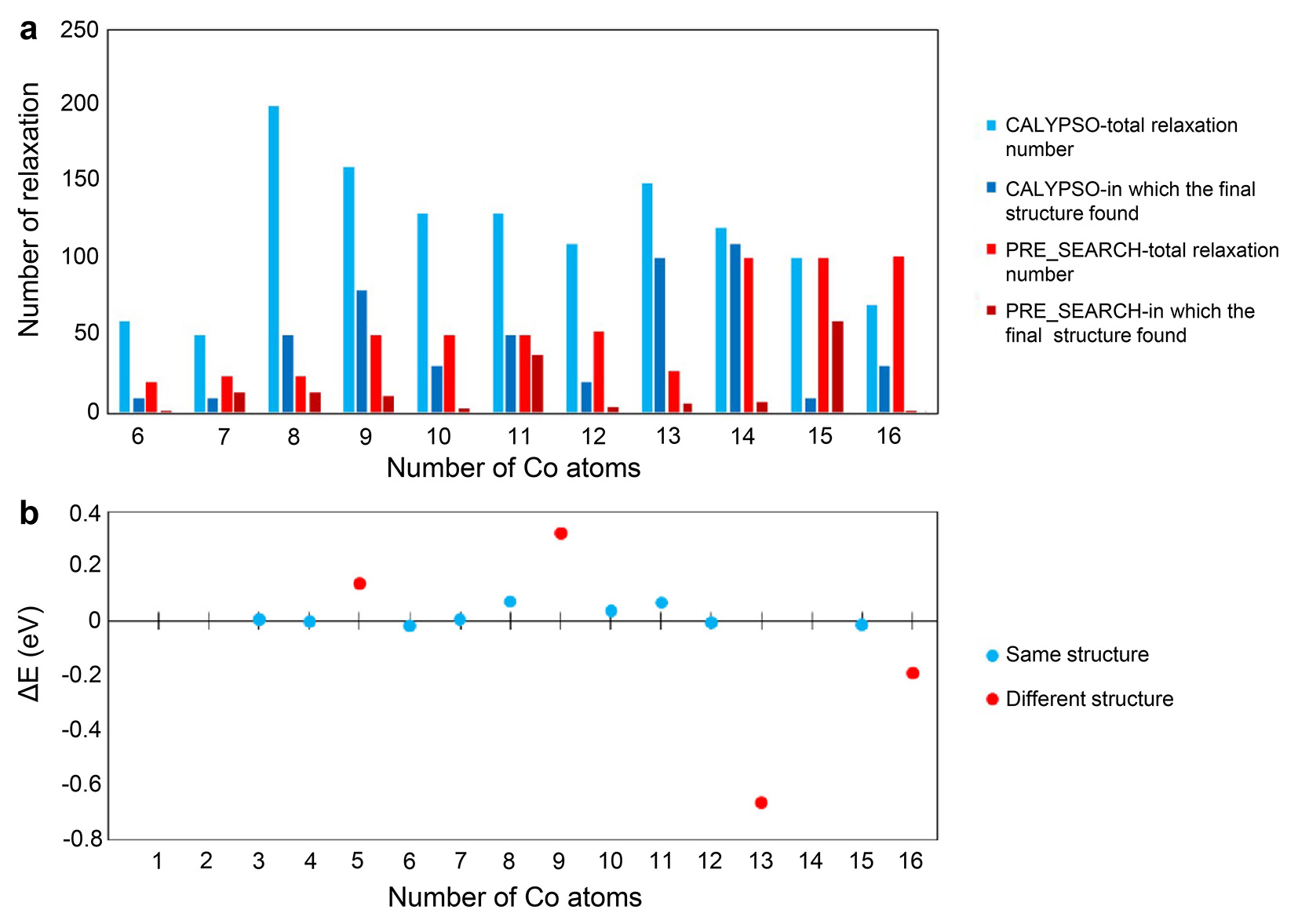


**Supplementary Figure 9.** Test of theoretical methods. A comparison of the structural search code CALYPSO with our pre-search procedure. **a**, The number of relaxations made in CALYPSO code and pre-search procedure. The light blue and red bars indicate the total searching number of relaxations. The number of total searching number can be manually changed. The dark blue and dark red bars indicate, in which relaxation the procedure finds its final energy minimum result. **b**, The energy difference between the final structure by CALYPSO search and our pre-search. As the two codes reach its final structures above, this graph shows whether the two structures are the same. $\Delta E$ is defined as the energy of $E_{pre-search}-E_{CALYPSO}$. Each point below 0 indicates a lower energy structure found by our pre-searching procedure, the points above 0 correspond to those with a lower energy structure by CALYPSO searching method. Red points mean different structures by the two procedures; while blue points indicate similar structures.

**Supplementary Table 1.** A comparison showing the total number of structures in searching for the energy minimum of Co_n_, by structural search code CALYPSO vs. pre-search procedure used in this study.

| n value | Total structures run by CALYPSO | The number for CALYPSO to find the energy minima | Total structures run by the pre-search method in this study | The number for the pre-search to find energy minima |
| --- | --- | --- | --- | --- |
| 3 | 60 | 31-40 | 4 | 2 |
| 4 | 20 | 1-10 | 11 | 4 |
| 5 | 90 | 51-60 | 10 | 1 |
| 6 | 60 | 1-10 | 20 | 1 |
| 7 | 50 | 1-10 | 23 | 13 |
| 8 | 200 | 41-50 | 23 | 13 |
| 9 | 160 | 71-80 | 50 | 11 |
| 10 | 130 | 21-30 | 50 | 2 |
| 11 | 130 | 41-50 | 50 | 38 |
| 12 | 110 | 11-20 | 53 | 4 |
| 13 | 150 | 91-100 | 27 | 6 |
| 14 | 120 | 101-110 | 100 | 7 |
| 15 | 100 | 1-10 | 101 | 60 |
| 16 | 70 | 21-30 | 102 | 1 |

*3.1.2 Basin-hopping global minimum search using TGMin code*

There are several previously published studies showing the electronic and geometric structures and bonding nature in such transition metal clusters and the oxides bridging the disciplines of physics and chemistry[40-68]. Based on these literatures, to more conclusively determine whether Co_13_O_8_ is truly a magic cluster of a cubic structure[69-71], we have also conducted basin-hopping global minimum search using TGMin code[72] to confirm the above calculations. TGMin is a self-developed program used for global minimum searching of geometric structures for gas-phase or surface-supported clusters, implemented with constrained basin-hopping (BH) algorithm. As results, we reproduced the global minimum structure of Co_13_O_8_ as found by the aforementioned method.

*3.1.3 The optimized lowest-energy structures*


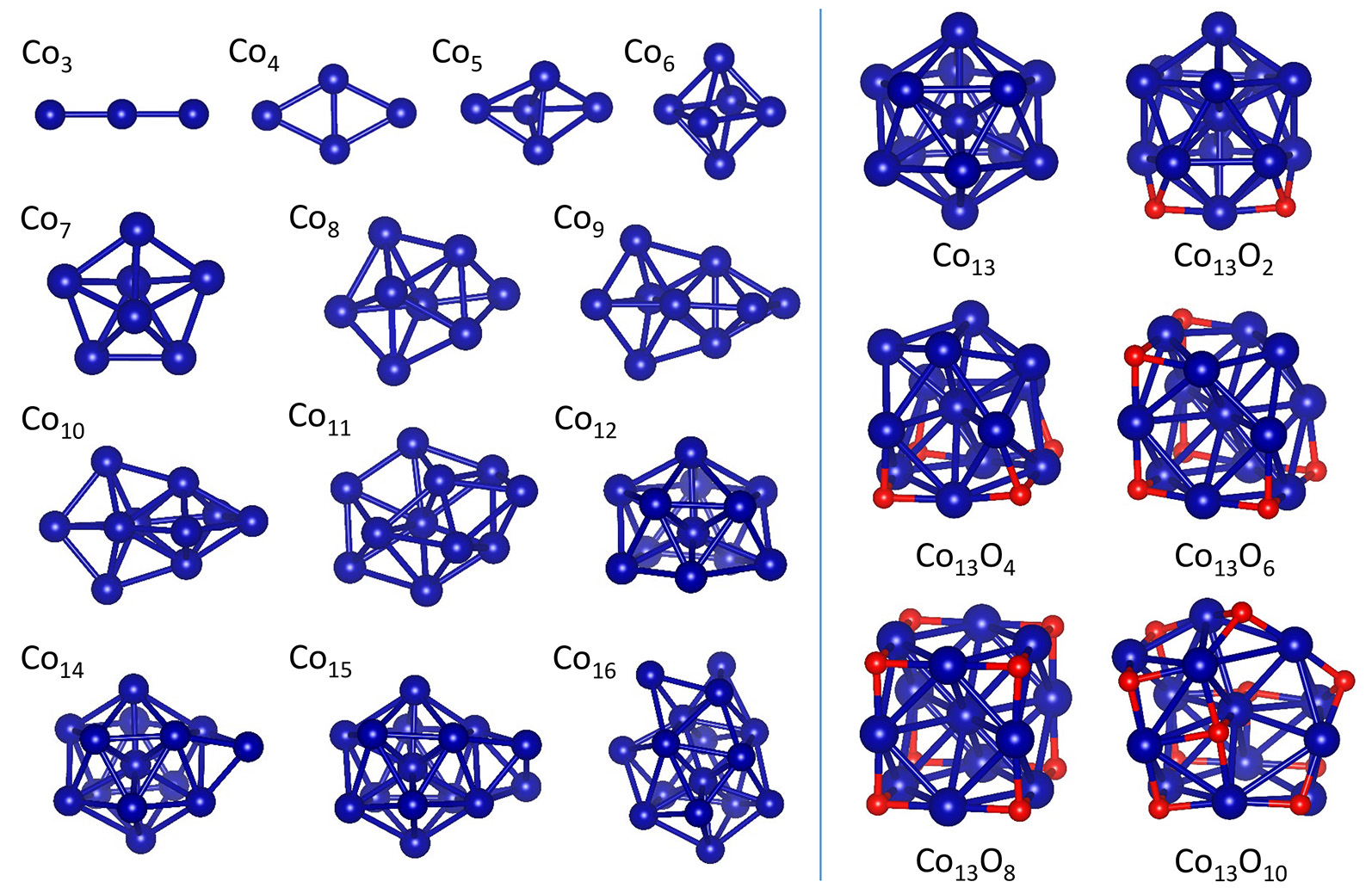


**Supplementary Figure 10.** The optimized lowest-energy structures of Co_n_ (n=3-16) and Co_13_O_m_ (m=2, 4, 6, 8,10).

**
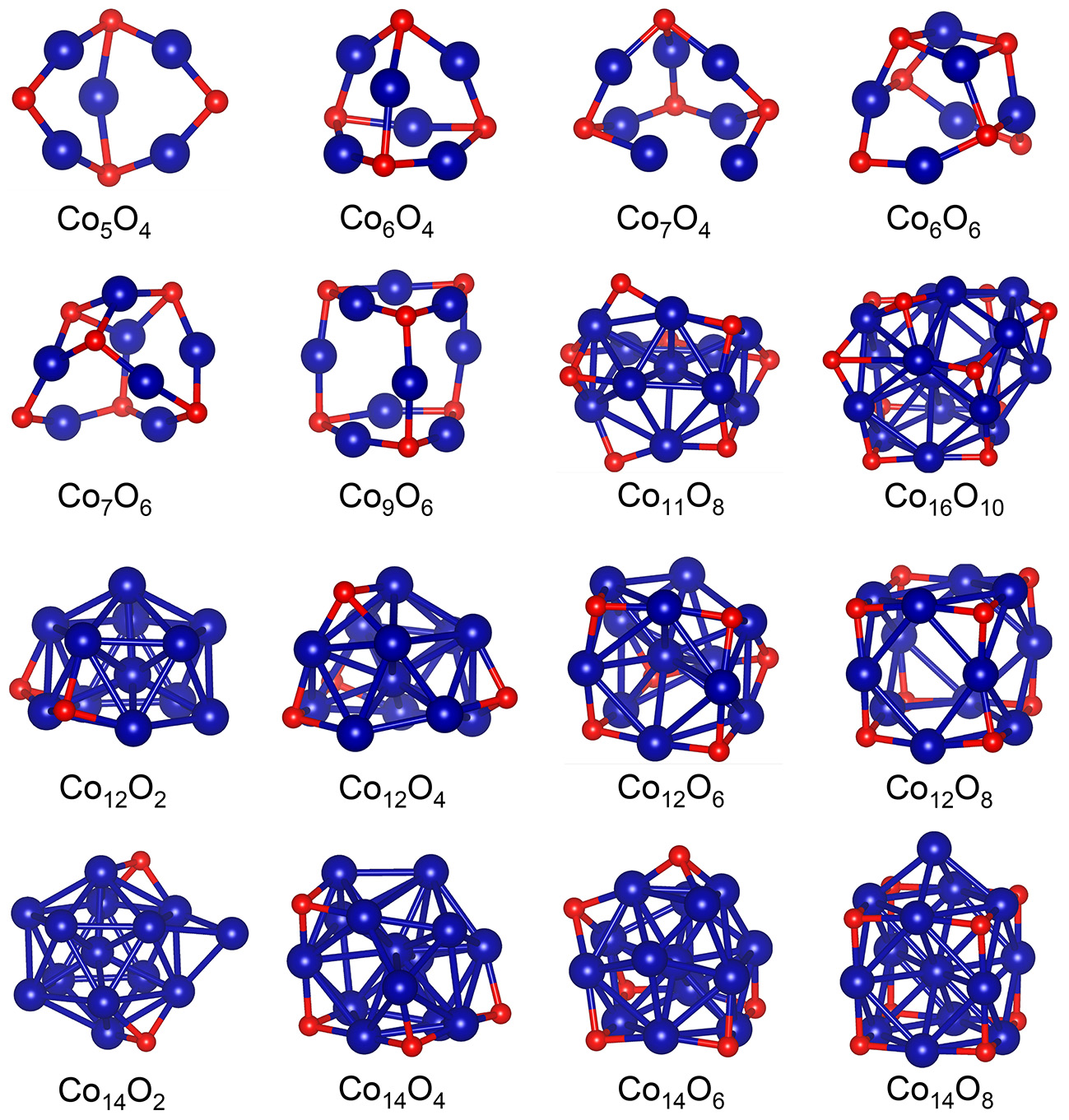
**

**Supplementary Figure 11.** The optimized structures of the other oxide clusters.

## Section 3.2 Energy calculation and thermodynamics

*3.2.1 Energy calculations and HOMO-LUMO gaps*

We performed the density functional theory calculations in PWmat code utilizing graphics processing unit (GPU) processors. NCPP-SG15-PBE pseudopotential with Perdew–Burke–Ernzerhof (PBE) exchange correlation functional is used in the energy calculations. As a basis set of plane wave used in PWmat code, a vacuum space of 20 Å in x, y and z direction was used to screen the coulomb interaction from the periodic boundaries. Considering that density functional theory (DFT) implemented with Kohn-Sham equations may not accurately describe strong-correlated systems, here we also employ Hubbard U method as a solution, for which the U value is given by experimental results [73, 74].


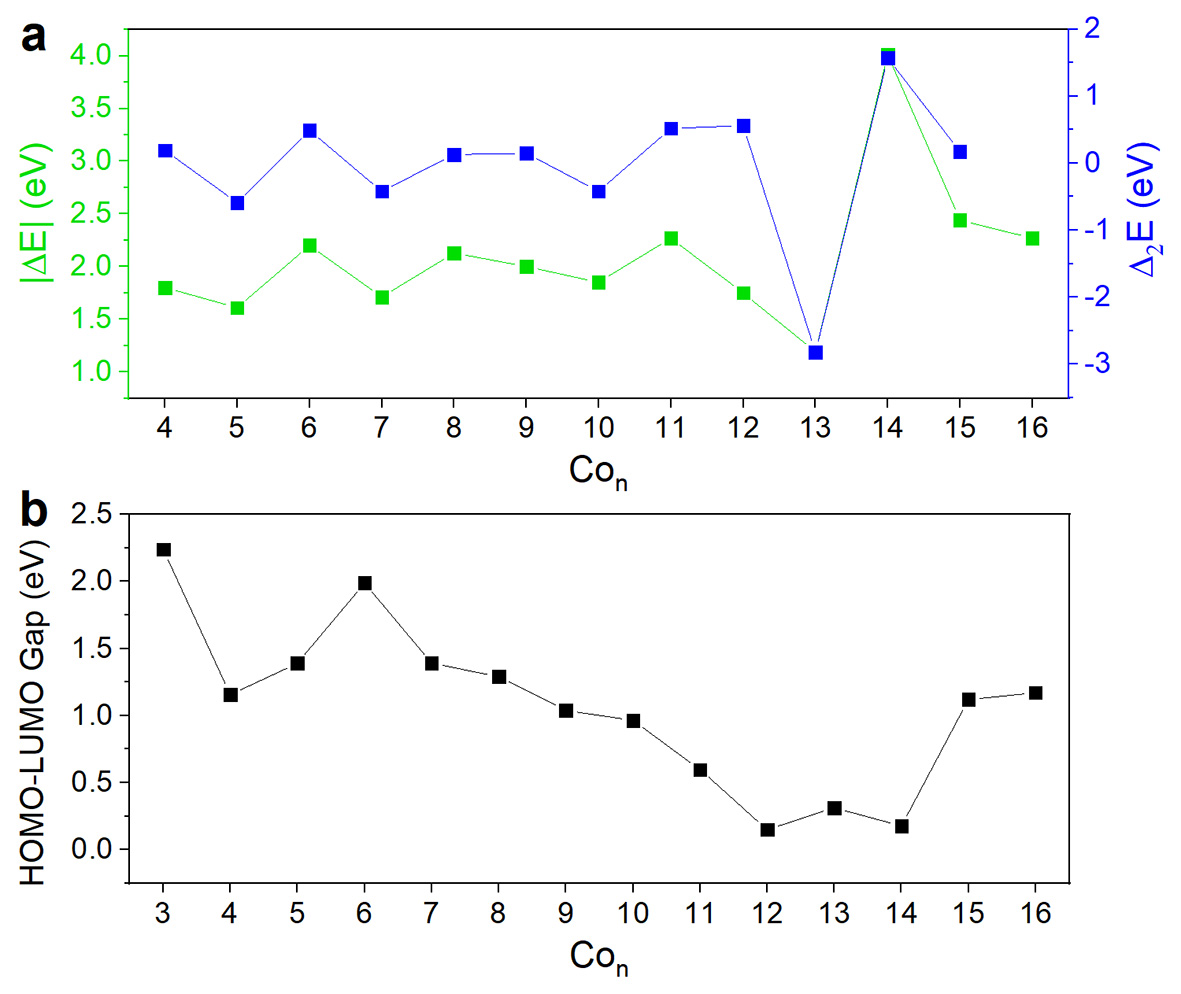


**Supplementary Figure 12.** DFT-calculated binding energy given by |∆E| = |E(Co_n_) – E(Co_n-1_) – E(Co)|, and ∆_2_E = E(Co_n-1_) + E(Co_n+1_) – 2E(Co_n_), as well as the DFT-calculated HOMO-LUMO gaps of the Co_n_ clusters.


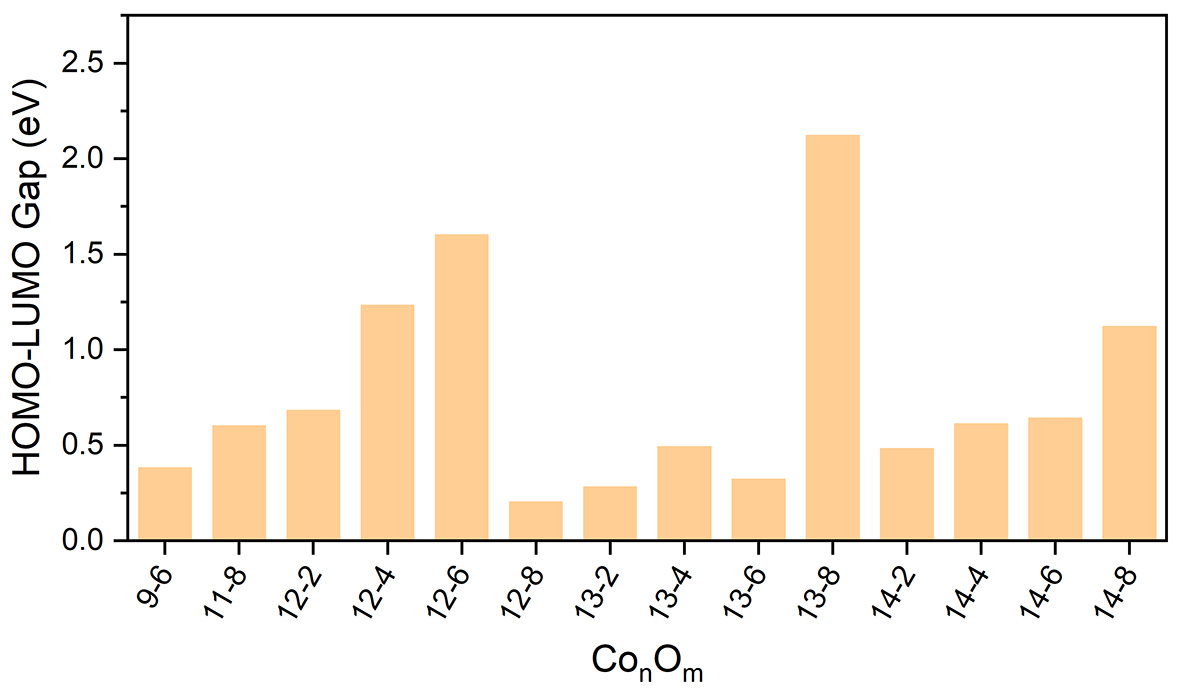


**Supplementary Figure 13.** DFT-calculated HOMO-LUMO gaps of the typical Co_n_O_m_ clusters.

## Section 3.3 Spin multiplicity

**Supplementary Table 2.** The varied spin multiplicity (2S+1) and relative energy of the Co_13_O_8_ cluster based on the pure BPW91 functional with 6-311G and 6-311G(3df) basis sets.

| **Spin multiplicity** | **Relative Energy (eV)** | |
| --- | --- | --- |
|  | 6-311G | 6-311G (3df) |
| 20 | 1.13 |  |
| 22 | 0.86 |  |
| 24 | 0.53 |  |
| 26 | 0.10 | 0.03 |
| 28 | 0.08 | 0.02 |
| **30** | **0** | **0** |
| 32 | 1.97 | 2.12 |
| 34 | 4.71 |  |
| 36 | 7.46 |  |

We also searched for the lowest-energy spin states of Co@Co_12_O_8_ using scalar-relativistic PBE/TZ2P with ADF code and confirmed the S=29/2 (2S+1=30) to be the lowest energy state, seen as Supplementary Fig. 14.

**
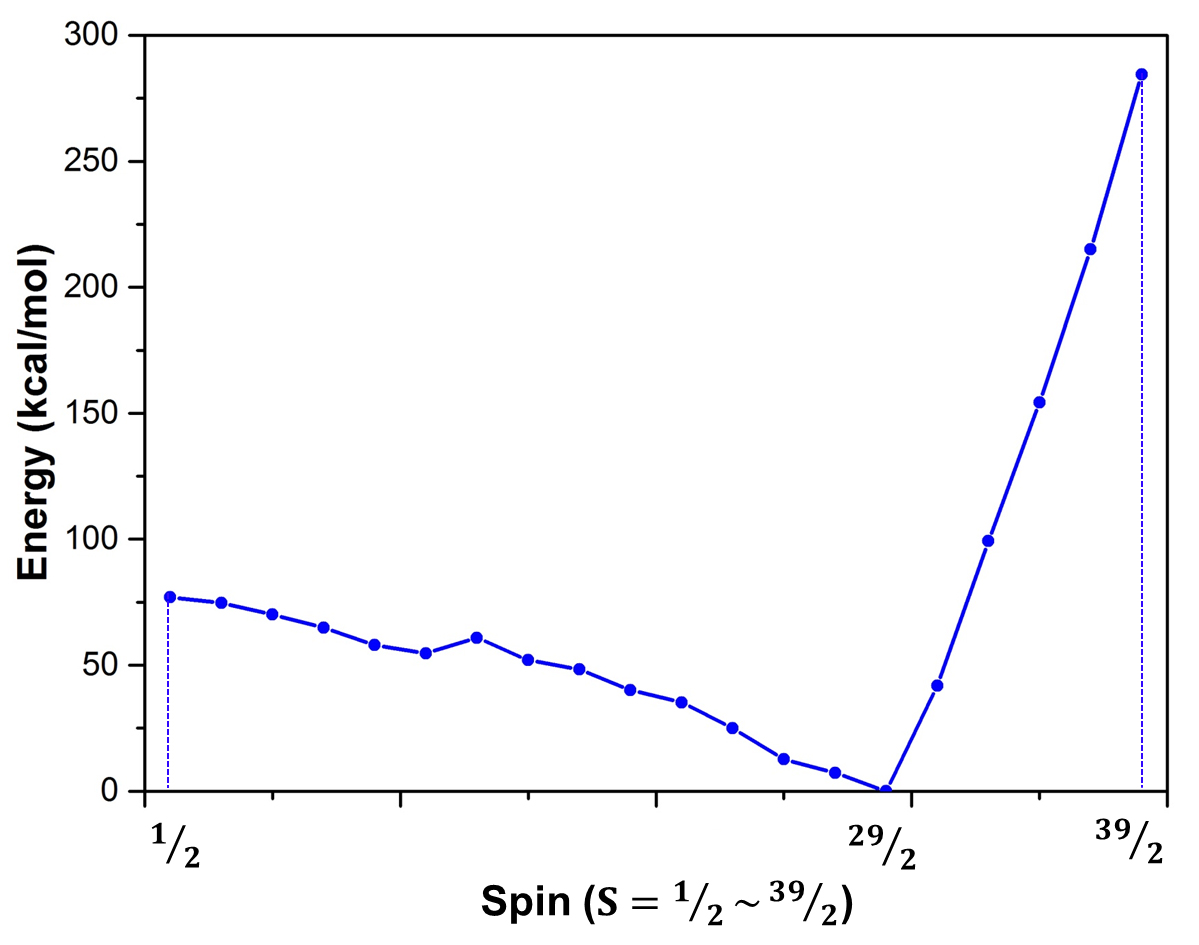
**

**Supplementary Figure 14.** The relationship of energy with spin quantum number (S=1/2 ~ 39/2). It is confirmed that the S=29/2 (2S+1=30) is the lowest energy spin state.

## Section 3.4 Dynamic structure transition in physical networks

Employing ab-initio molecular dynamic (AIMD) simulation[75], we have studied the structures in a Langevin-NVT system within different temperatures[76]. The time interval for each step was set to be 2 fs, and a relaxation with a residue force less than 0.005 eV/Å is made. The Hubbard U value was set to be 3.32 eV, same as the aforementioned DFT calculations. On this basis, we have firstly tested the stability of Co_13_O_8_ cluster. Supplementary Table 3 shows the stability of Co_13_O_8_ cluster within molecular dynamics simulation of 2000 fs. It was found that, at 300 K, Co_13_O_8_ cluster survive two additional oxygen molecules attacking; at 600 K and even 900 K, it is still free of dissociation at the presence of two additional oxygen molecules. Remarkably, Co_13_O_8_ retains the cubic structure at 1200 K and is inert even at the presence of an additional oxygen molecule. In comparison, Co_13_O_6_ is much less stable at the presence of oxygen.

**Supplementary Table 3.** The dynamic stability of Co_13_O_8_ cluster. Dynamic simulations are conducted by setting additional oxygen around the Co_13_O_8_ cluster in different temperature, bcc-stable refers to the unaffected body-centered cubic structure.

| Temperature | 300 K | 600 K | 900 K | 1200 K |
| --- | --- | --- | --- | --- |
| **Co_13_O_8_ + O_2_** | bcc-stable | bcc-stable | bcc-stable | bcc-stable |
| **Co_13_O_8_ + 2 O_2_** | bcc-stable | bcc-stable | bcc-stable | bcc-dissociative |

**Supplementary Table 4.** The dynamic stability of Co_13_O_6_ cluster. Dynamic simulations are conducted by setting additional oxygen around the Co_13_O_6_ cluster.

| Temperature | 300 K | 600 K | 900 K |
| --- | --- | --- | --- |
| **Co_13_O_6_ + O_2_** | reactive | reactive | reactive |

## Section 3.5 Reaction dynamics calculations

*3.4.1 Chemical reaction kinetics for “Co_13_ + O_2_”*

Employing DFT calculations based on Gaussian 16 software package[77], we have further calculated the energy diagram to address the reaction kinetics by assuming an oxygen molecule attacks a Co_13_ cluster from different orientations. The spin-triplet ^3^O_2_ reacts with Co_13_ via an “end-on” attack orientation (^28^IM1, i.e., a superoxide state)[78, 79], and a transformation to peroxide state (^28^IM2) is readily attained with a neglectable transition state barrier of 0.04 eV (TS1). Following that, the reaction pathway is barrierless towards the formation of Co_13_O_2_ with dissociative O-O bond. Note that, the supposed transition state (^28^TS2) for the O-O bond dissociation is completely submerged by the structural relaxation of the cluster, with 0.01 eV lower in energy than that of the peroxide state (^28^IM2) when the zero-point correction energy (ZPE) is taken into consideration[80, 81]. Subsequently, under spin conservation or inversion[27], the ^28^IM2 state could form a dissociated state ^28^P (-7.31 eV) or a more stable ^26^P state (-7.52 eV). Besides, we have also considered the oxygen molecule attaching a Co_13_ cluster via other orientations, but found the same local minimum structure with O-O bond dissociation at the optimization process.

*3.4.2 DFT-calculated structures for “Co_13_O_2X_ + O_2_”*

Considering the diversity of “Co_13_O_2X_ + O_2_”, we calculated the typical adsorption structures to show O-O bonds stretched by the adsorption of oxygen molecules on the Co_13_O_2x_ (x=1-4) clusters, as shown in Supplementary Fig. 15. When an O_2_ molecule horizontally attached to the triangular surface of Co_13_O_2,4,6_ clusters, the O-O bond length is the longest (~ 1.50 Å) among them.


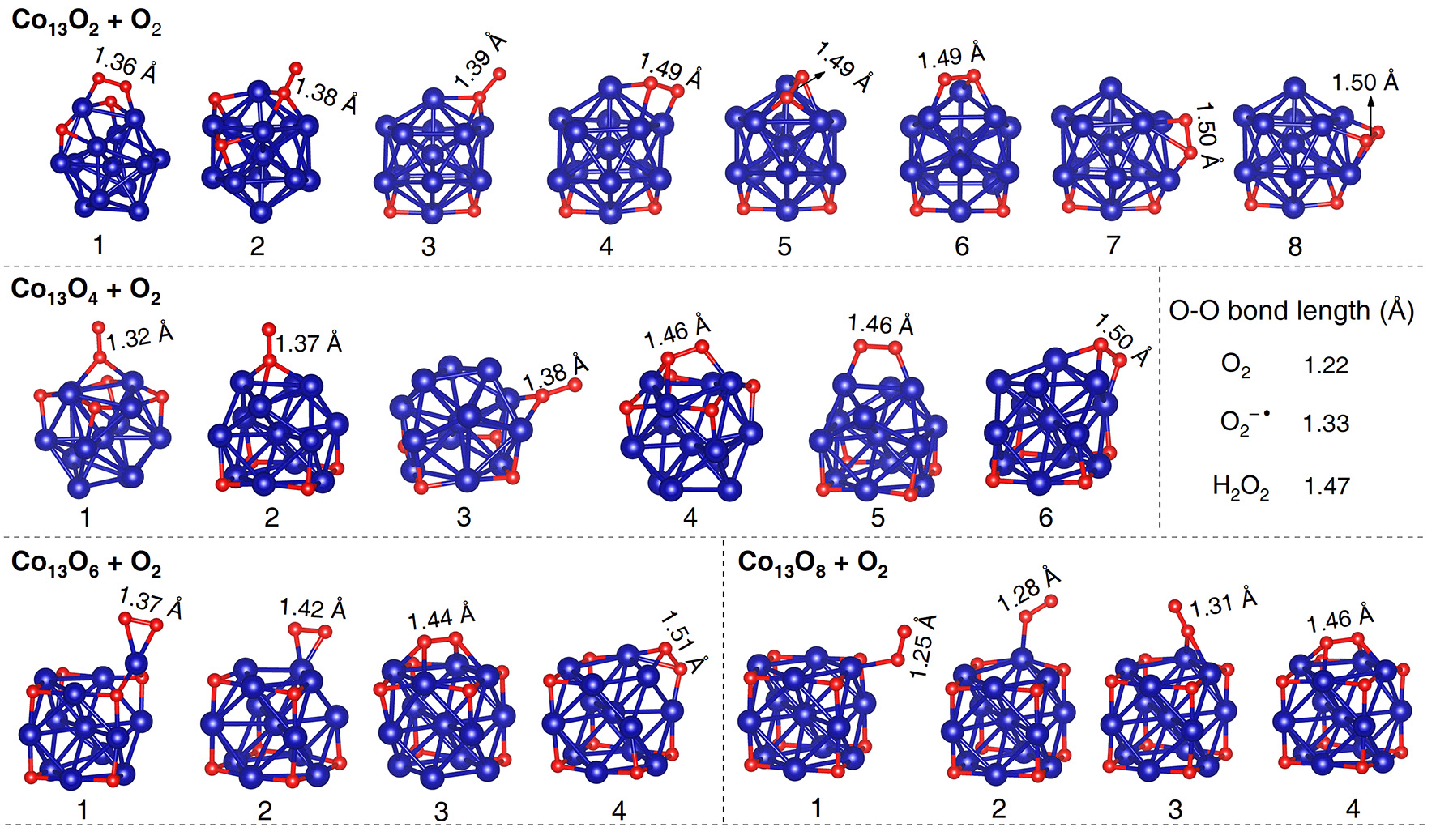


**Supplementary Figure 15.** Optimized typical structures of diverse adsorption of O_2_ on Co_13_O_2x_ (x=1-4) clusters, and the altered O-O bond lengths.

It is worth noting that the absolute value of maximum binding energy of O_2_ adsorption on Co_13_O_8_ is much lower than all the other Co_13_O_2x_ (x=1-3) clusters, which reveals that Co_13_O_8_ has a lower activity to bind additional oxygen than the other Co_13_O_2x_ (x=1-3) clusters.


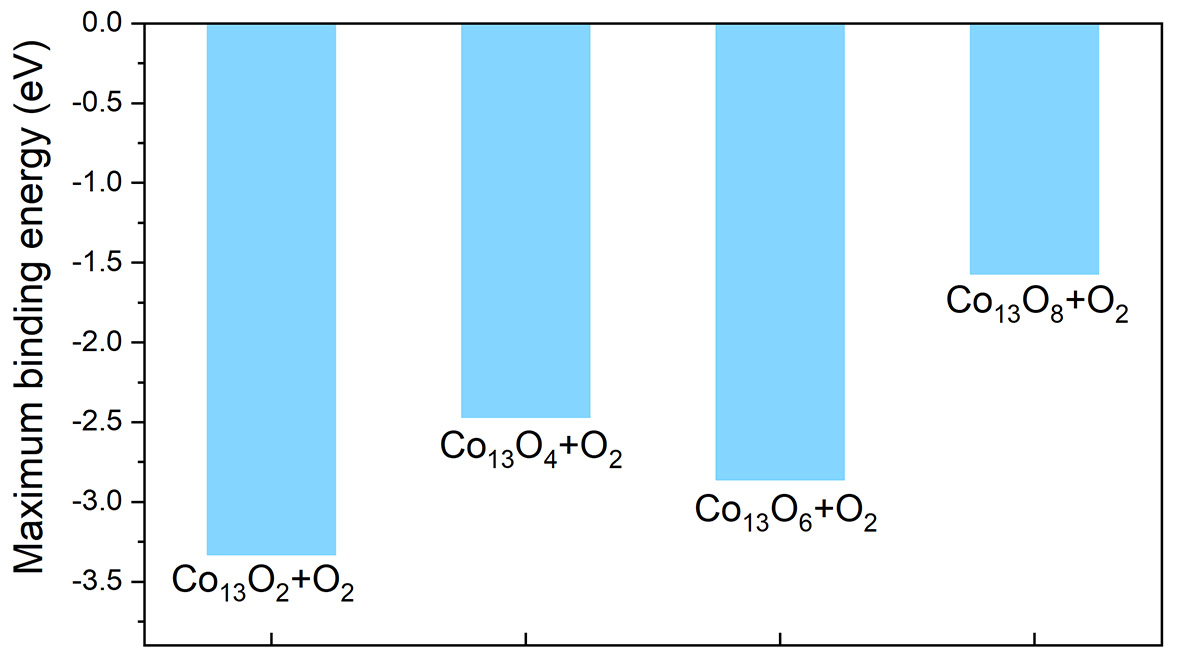


**Supplementary Figure 16.** Maximum binding energies of O_2_ on Co_13_O_2x_ (x=1-4) clusters corresponding to the structures in supplementary Fig. 15. The binding energy is defined as E_b_ = E(Co_13_O_2x+2_) – E(Co_13_O_2x_) – E(O_2_).

**Supplementary Table 5.** Typical adsorption structures of O_2_ on Co_13_O_2x_ (x=1-3) clusters, with O-O bond lengths and binding energies corresponding to the structures shown in Supplementary Fig. 15. The binding energy is defined as E_b_ = E(Co_13_O_2x+2_) – E(Co_13_O_2x_) – E(O_2_).

|  | **Structures of O_2_ on Co_13_O_2x_ clusters** | **O-O bond length (Å)** | **E_b_ (eV)** |
| --- | --- | --- | --- |
| Co_13_O_2_ + O_2_ | Structure 1 | 1.36 | -1.22 |
|  | Structure 2 | 1.38 | -1.56 |
|  | Structure 3 | 1.39 | -1.46 |
|  | Structure 4 | 1.49 | -1.97 |
|  | Structure 5 | 1.49 | -2.78 |
|  | Structure 6 | 1.49 | -2.75 |
|  | Structure 7 | 1.50 | -3.18 |
|  | Structure 8 | 1.50 | **-3.33** |
| Co_13_O_4_ + O_2_ | Structure 1 | 1.32 | -0.24 |
|  | Structure 2 | 1.37 | -0.30 |
|  | Structure 3 | 1.38 | -0.85 |
|  | Structure 4 | 1.46 | -1.22 |
|  | Structure 5 | 1.46 | -2.01 |
|  | Structure 6 | 1.50 | **-2.47** |
| Co_13_O_6_ + O_2_ | Structure 1 | 1.37 | -0.49 |
|  | Structure 2 | 1.42 | -1.45 |
|  | Structure 3 | 1.44 | -0.95 |
|  | Structure 4 | 1.51 | **-2.86** |
| Co_13_O_8_ + O_2_ | Structure 1 | 1.25 | 0.22 |
|  | Structure 2 | 1.28 | -0.36 |
|  | Structure 3 | 1.31 | -0.36 |
|  | Structure 4 | 1.46 | **-1.57** |

## Section 3.6 NICS and spin density population

Quantum chemical studies were also conducted using relativistic density functional theory (DFT) implemented in the Amsterdam Density Functional (ADF) program [82-84]. The generalized gradient approximation (GGA) with the PBE exchange-correlation functional[85] and hybrid meta-GGA functional M06-2X were used [86, 87], together with the uncontracted TZ2P Slater basis sets for all the atoms[88]. Frozen core approximations were applied to the inner shells [1s^2^-2p^6^] for Co and [1s^2^] for O atoms. The scalar relativistic (SR) effects were considered by the zero-order-regular approximation (ZORA) to account for the mass-velocity and Darwin effects[89]. The atomic net charges were evaluated using the partition of Mulliken [90], Hirshfeld [91], Voronoi deformation density (VDD) [92], and MDC-q schemes [93]. Nucleus-independent chemical shifts (NICS) [94-97] values were calculated at the center of the Co_4_O_4_ surface (NICS(0)) and 1.0 Å above the surface (NICS(1)) using all-electron Slater basis sets, and these values were used as criteria to evaluate the cubic aromaticity of this cluster.


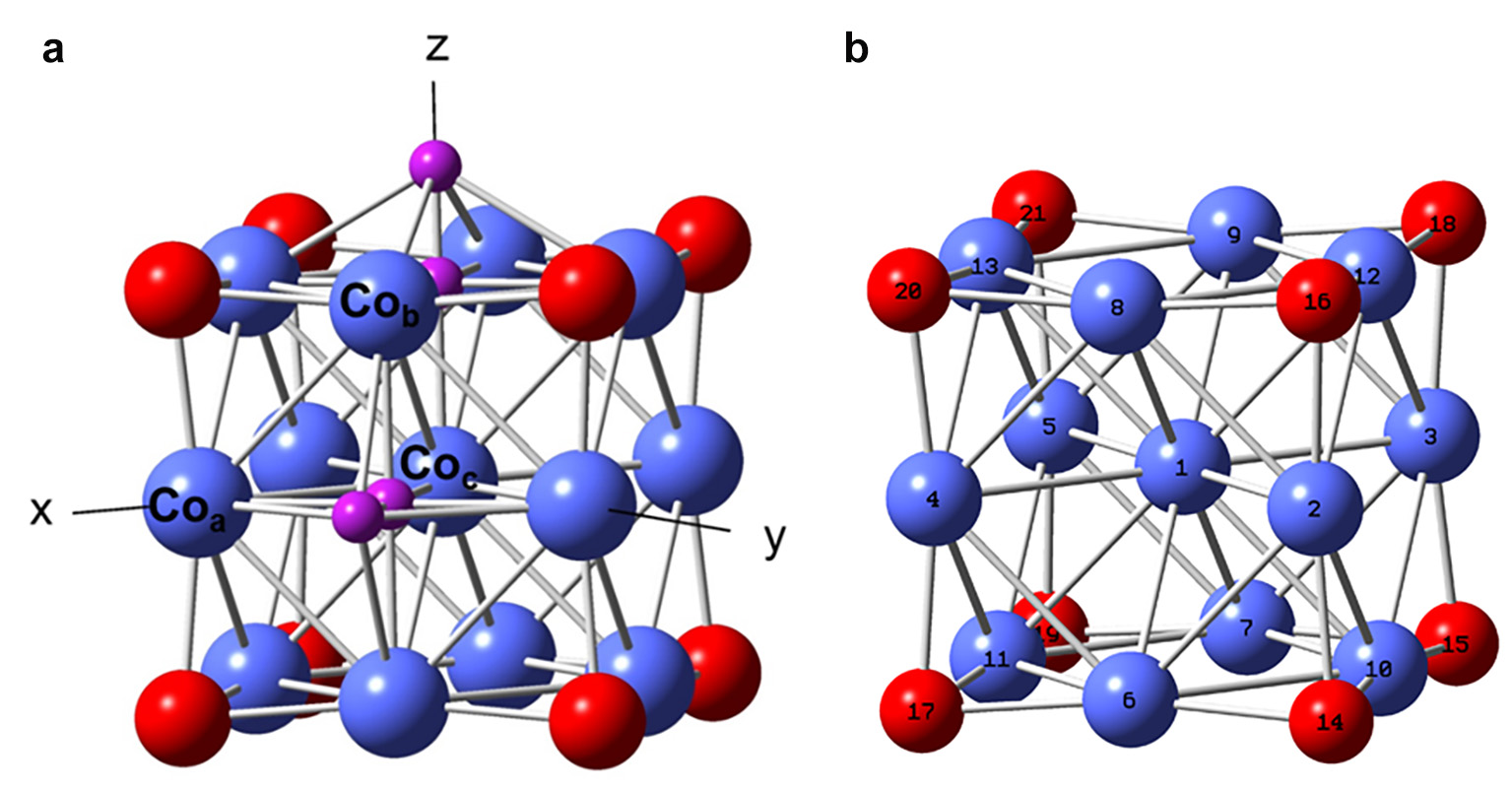


**Supplementary Figure 17.** Optimized geometric structure of *D_4h_*-Co@Co_12_@O_8_ cluster with ghost atoms lying at the center of the Co_4_O_4_ surface and 1.0 Å above the surface. Color code: Co (blue), O (red), dummy atom (purple).

**Supplementary Table 6.** NICS values (in ppm) of Co_13_O_8_, benzene and Zn^I^_8_ calculated at the center of the Co_4_O_4_ surface of the pseudo-cubic cluster (NICS(0)) and at 1.0 Å above the center of the surface (NICS(1)) at the SR-ZORA PBE/TZ2P level of theory.

|  | **Atom** | **NICS(0)** | **NICS(1)** |
| --- | --- | --- | --- |
| **Co_13_O_8_** | Bq_z_*^a^* | -54.0 | -22.0 |
|  | Bq_xy_*^a^* | -54.0 | -21.9 |
| **Zn^I^_8_*^b^*** | Bq_z_*^a^* | -28.0 | -6.4 |
|  | Bq_xy_*^a^* | -28.3 | -6.9 |
| **benzene** | Bq_z_*^a^* | -7.4 | -9.7 |

*^a^* Bq_z_ represents the ghost atom along the z axis while Bq_xy_ the ghost atom in the xy plane. *^b^* NICS values of Zn^I^_8_ reported in literature [98].

**Supplementary Table 7.** The calculated spin density population and net charges of atoms in Co_13_O_8_ cluster at the SR-ZORA M06-2X/TZ2P level of theory.

| Atom*^a^* | Spin | Mulliken | Hirshfeld | VDD | MDC-q |
| --- | --- | --- | --- | --- | --- |
| Co_c_ | 1.51 | -0.43 | -0.09 | -0.11 | -0.06 |
| Co_a_ | 2.10 | 0.60 | 0.20 | 0.20 | 0.69 |
| Co_b_ | 2.34 | 0.71 | 0.30 | 0.32 | 0.66 |
| O | 0.04 | -0.96 | -0.39 | -0.40 | -1.00 |

*^a^* Co_c_ represents Co atom at the center of the cluster (i.e., Co@Co_12_@O_8_). Co_a_ represents Co atom in the xy plane while Co_b_ represents Co atom above or below the xy plane.

## Section 3.7 The bond lengths and natural population analysis

**Supplementary Table 8.** The bond lengths (Å) of Co-Co and Co-O in Co_13_O_8_ cluster.

| Co_13_O_8_ | Bond length |
| --- | --- |
| Co_a_-Co_b_ | 2.40 |
| Co_a_-Co_c_ | 2.40 |
| Co_a_-O | 1.83 |

**Supplementary Table 9.** The charge distribution of Co_13_O_8_ cluster by natural population analysis.

| Atom | No. | Natural Charge | Classification |
| --- | --- | --- | --- |
| Co | 1 | -0.13814 | Co_c_ |
| Co | 2 | 0.71378 | Co_a_  Co_a_  Co_a_  Co_a_ |
| Co | 3 | 0.71378 |  |
| Co | 4 | 0.71378 |  |
| Co | 5 | 0.71378 |  |
| Co | 6 | 0.71378 | Co_b_ |
| Co | 7 | 0.71378 | Co_b_ |
| Co | 8 | 0.71378 | Co_b_ |
| Co | 9 | 0.71378 | Co_b_ |
| Co | 10 | 0.71378 | Co_b_ |
| Co | 11 | 0.71378 | Co_b_ |
| Co | 12 | 0.71378 | Co_b_ |
| Co | 13 | 0.71378 | Co_b_ |
| O | 14 | -1.05404 |  |
| O | 15 | -1.05404 |  |
| O | 16 | -1.05404 |  |
| O | 17 | -1.05404 |  |
| O | 18 | -1.05404 |  |
| O | 19 | -1.05404 |  |
| O | 20 | -1.05404 |  |
| O | 21 | -1.05404 |  |
| Total | * | 0.00000 |  |

## Section 3.8 Molecular orbital analysis

To gain insights into the bonding nature of the Co_13_O_8_ cluster and unravel how it possesses delocalized π bonds in six faces of the cubic lattice structure, we performed systematic molecular orbital (MO) analysis. Supplementary Fig. 18 shows a few occupied MOs which provide the main contributions to the aromaticity of this cluster. The program packages of Multiwfn[99] and VMD[100] software were used to draw the figures.


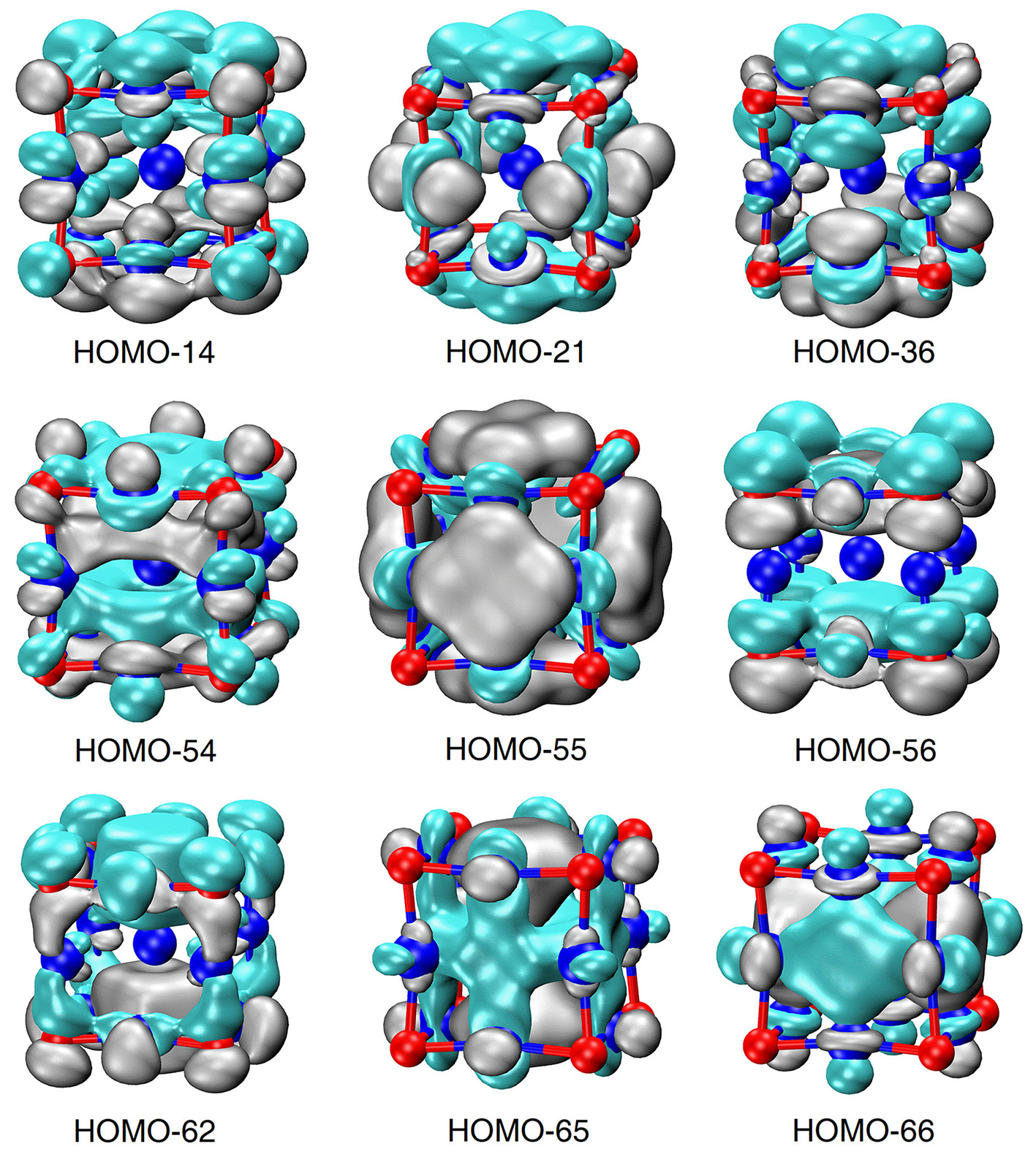


**Supplementary Figure 18.** Orbital analysis. A few typical occupied orbitals of the Co_13_O_8_ cluster corresponding to electron delocalization.

## Section 3.9 Ionization energies of Co_n_O_m_ clusters

**Supplementary Table 10.** The vertical ionization energies (VIE) of Co_n_O_m_ (n=12-14, m=2-8).

| **Co_n_O_m_** | **Spin multiplicity** | **VIE (eV)** |
| --- | --- | --- |
| Co_12_O_2_ | 25 | 5.52 |
| Co_12_O_4_ | 27 | 6.10 |
| Co_12_O_6_ | 27 | 6.36 |
| Co_12_O_8_ | 17 | 6.65 |
| Co_13_O_2_ | 26 | 5.52 |
| Co_13_O_4_ | 26 | 5.84 |
| Co_13_O_6_ | 26 | 6.33 |
| Co_13_O_8_ | 30 | 6.89 |
| Co_13_O_10_ | 34 | 6.94 |
| Co_14_O_2_ | 29 | 5.50 |
| Co_14_O_4_ | 29 | 5.83 |
| Co_14_O_6_ | 29 | 6.09 |
| Co_14_O_8_ | 31 | 6.57 |

**Supplementary Table 11.** Ionization Energies (IEs) of the other Co_n_O_m_ clusters. The IEs of Co_n_O_m_ clusters (n=1-8, n≥m), collected from the previously published studies[68, 101].

| Co_n_O_m_ isomer | Neutral/cation | | Ionization energies (eV) |
| --- | --- | --- | --- |
|  | **Symmetry** | **Magnetic moment (μ_B_)** |  |
| CoO |  | 3/4 | 8.76 |
| CoO_2_ |  | 1/2 | 10.05 |
| Co_2_O |  | 4/5 | 7.49 |
| Co_2_O_2_ |  | 0/5 | 7.9 |
| Co_3_O-I | C_3v_ | 7/6 | 6.54 |
| Co_3_O-II | C_s_ | 7/8 | 7.25 |
| Co_3_O_2_-I | C_2v_ | 9/8 | 7.28 |
| Co_3_O_2_-II | C_s_ | 7/8 | 7.02 |
| Co_3_O_3_-I | C_2v_/D_3h_ | 9/10 | 8.35 |
| Co_3_O_3_-II | C_∞ v_ | 9/10 | 7.79 |
| Co_4_O-I | C_s_/C_3v_ | 8/9 | 6.42 |
| Co_4_O-II | C_2v_/C_s_ | 8/9 | 6.79 |
| Co_4_O_2_-I | C_2v_/C_3v_ | 10/9 | 6.90 |
| Co_4_O_2_-II | C_s_ | 10/11 | 7.67 |
| Co_4_O_3_-I | C_s_ | 8/11 | 7.23 |
| Co_4_O_3_-II | C_s_ | 10/11 | 7.49 |
| Co_4_O_4_-I | D_4h_ | 12/13 | 8.54 |
| Co_4_O_4_-II | C_s_ | 10/11 | 8.01 |
| Co_4_O_4_-III | T_d_ | 12/13 | 7.52 |
| Co_5_O-I | C_s_ | 11/10 | 6.44 |
| Co_5_O-II | C_s_ | 11/10 | 6.59 |
| Co_5_O-III | C_s_ | 11/10 | 6.70 |
| Co_5_O_2_-I | C_1_ | 11/12 | 6.98 |
| Co_5_O_2_-II | C_1_ | 11/12 | 7.01 |
| Co_5_O_3_-I | C_s_ | 13/12 | 7.55 |
| Co_5_O_3_-II | C_s_/C_1_ | 13/14 | 7.72 |
| Co_5_O_4_-I | C_2v_ | 13/14 | 8.13 |
| Co_5_O_4_-II | C_s_/C_1_ | 11/10 | 7.94 |
| Co_5_O_5_-I | C_s_ | 11/12 | 8.35 |
| Co_5_O_5_-II | C_1_/C_2v_ | 15/2 | 7.80 |
| Co_5_O_5_-III | C_s_ | 11/12 | 8.30 |
| Co_6_O-I | C_3v_ | 14/13 | 6.87 |
| Co_6_O-II | C_2v_ | 14/13 | 6.89 |
| Co_6_O_2_-I | C_2v_ | 14/13 | 6.94 |
| Co_6_O_3_-I | C_3v_ | 14/15 | 7.28 |
| Co_6_O_4_-I | T_d_ | 14/13 | 8.06 |
| Co_6_O_4_-II | D_2h_ | 14/15 | 7.22 |
| Co_6_O_5_-I | C_s_ | 16/15 | 7.83 |
| Co_6_O_5_-II | C_s_ | 16/17 | 7.87 |
| Co_6_O_5_-III | C_3v_ | 14/15 | 7.99 |
| Co_6_O_6_-I | C_2v_ | 14/19 | 7.40 |
| Co_6_O_6_-II | C_2v_ | 12/15 | 7.31 |
| Co_6_O_6_-III | D_3h_/C_2h_ | 12/15 | 8.15 |
| Co_7_O-I | C_s_ | 15/16 | 6.53 |
| Co_7_O-II | C_3v_ | 17/16 | 6.68 |
| Co_7_O_2_-I | C_s_ | 15/16 | 6.79 |
| Co_7_O_2_-II | C_1_ | 15/16 | 6.75 |
| Co_7_O_3_-I | C_3v_ | 15/16 | 7.29 |
| Co_7_O_4_-I | C_s_ | 15/16 | 7.09 |
| Co_7_O_4_-II | C_s_ | 15/16 | 7.32 |
| Co_7_O_5_-I | C_s_ | 15/18 | 7.12 |
| Co_7_O_5_-II | C_s_ | 15/16 | 7.34 |
| Co_7_O_6_-I | C_3v_ | 11/12 | 7.89 |
| Co_7_O_6_-II | C_3v_ | 15/18 | 7.50 |
| Co_7_O_7_-I | C_s_ | 15/12 | 7.53 |
| Co_7_O_7_-II | C_s_ | 11/12 | 7.73 |
| Co_7_O_7_-III | C_3v_ | 11/12 | 7.45 |
| Co_8_O-I | C_1_ | 16/17 | 6.29 |
| Co_8_O-II | C_s_ | 18/19 | 6.55 |
| Co_8_O-III | C_2v_ | 18/19 | 6.77 |
| Co_8_O_2_-I | C_1_ | 16/17 | 6.41 |
| Co_8_O_2_-II | C_s_ | 18/17 | 6.47 |
| Co_8_O_2_-III | C_s_ | 18/19 | 7.07 |
| Co_8_O_3_-I | C_s_ | 16/17 | 6.84 |
| Co_8_O_3_-II | C_s_ | 18/19 | 6.94 |
| Co_8_O_3_-III | C_2v_ | 18/17 | 7.35 |
| Co_8_O_4_-I | C_2v_ | 18/19 | 7.19 |
| Co_8_O_4_-II | C_s_ | 16/17 | 6.84 |
| Co_8_O_4_-III | C_2_ | 16/17 | 7.16 |
| Co_8_O_5_-I | C_4v_ | 18/19 | 7.26 |
| Co_8_O_5_-II | C_1_ | 18/19 | 7.42 |
| Co_8_O_5_-III | C_2v_ | 20/21 | 7.10 |
| Co_8_O_6_-I | C_2v_/C_4v_ | 20/17 | 7.18 |
| Co_8_O_6_-II | C_2_ | 20/17 | 8.69 |
| Co_8_O_7_-I | C_s_ | 20/21 | 7.32 |
| Co_8_O_7_-II | C_1_ | 18/19 | 7.35 |
| Co_8_O_7_-III | C_1_ | 20/21 | 7.63 |
| Co_8_O_8_-I | C_2v_ | 22/19 | 7.84 |
| Co_8_O_8_-II | C_2_/C_1_ | 8/17 | 7.63 |
| Co_8_O_8_-III | C_s_ | 16/15 | 7.72 |

## Section 3.10 Co_13_O_8_ ring current

The gauge including magnetically induced current (GIMIC)[102, 103] is calculated utilizing the software package of GIMIC 2.1.4 at BPW91/6-311G(3df) level.


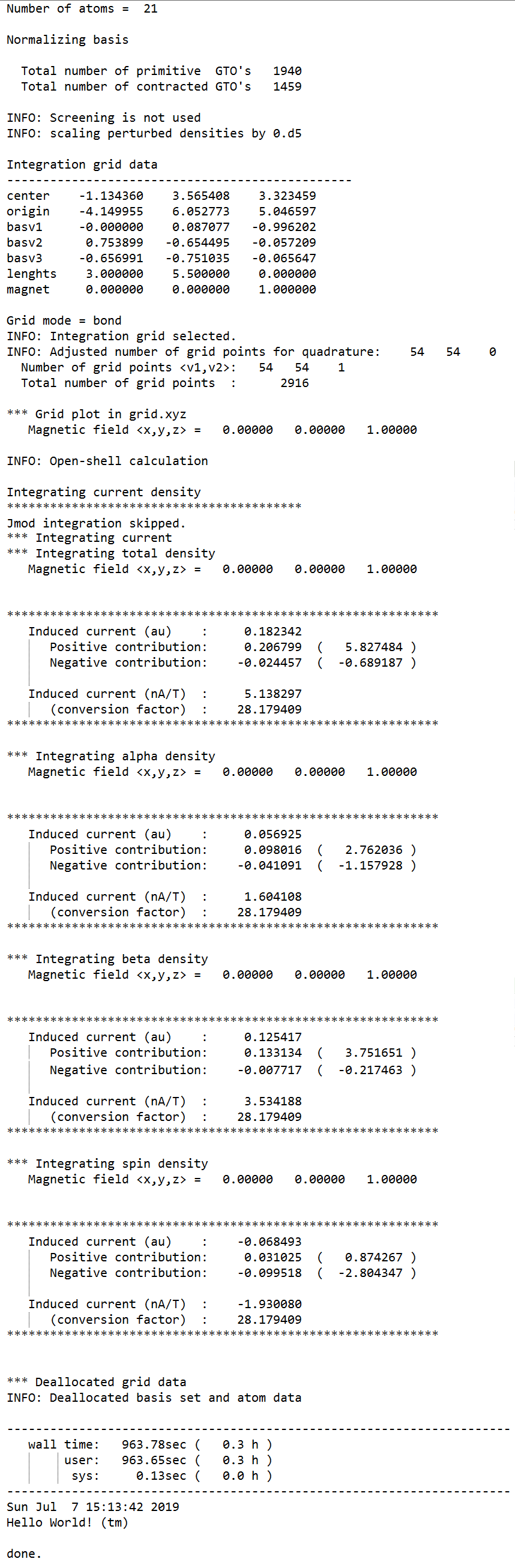


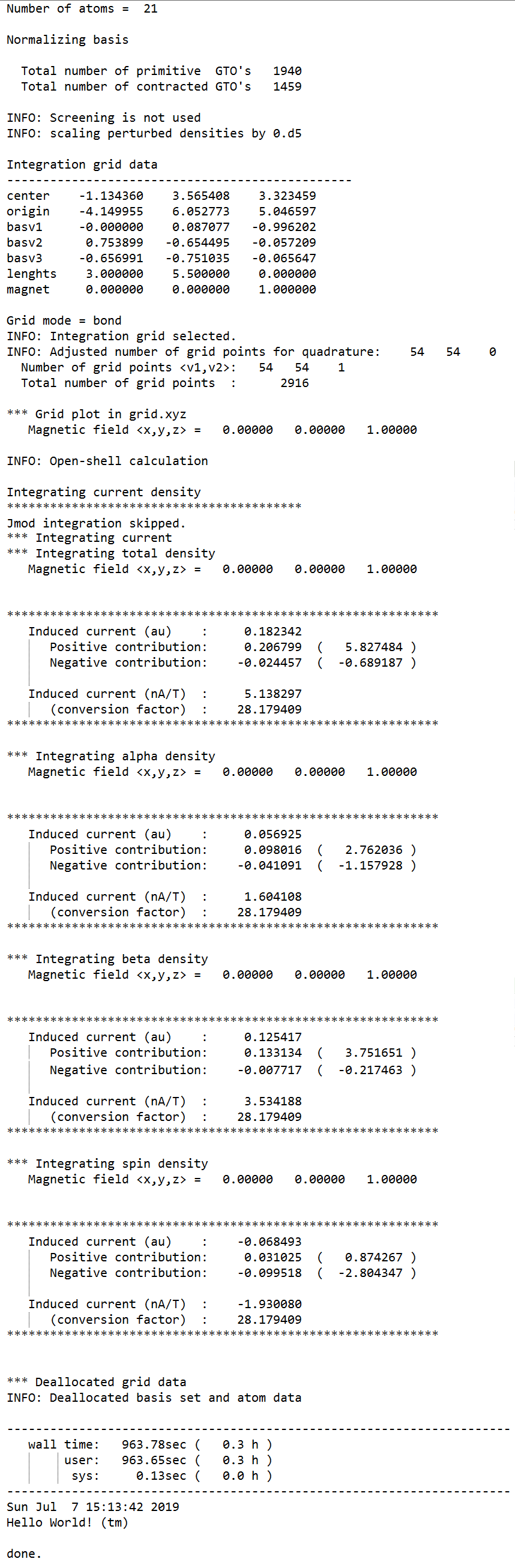


# References

1. Knudtson, JT, Eyring, EM. Laser-induced chemical reactions. *Annu Rev Phys Chem* 1974; **25**: 255-74.

2. Dietz, TG, Duncan, MA, Powers, DE *et al.* Laser production of supersonic metal cluster beams. *J Chem Phys* 1981; **74**: 6511-2.

3. Bondybey, VE, English, JH. Laser excitation spectra and lifetimes of Pb_2_ and Sn_2_ produced by YAG laser vaporization. *J Chem Phys* 1982; **76**: 2165-70.

4. Hopkins, JB, Langridge‐Smith, PRR, Morse, MD *et al.* Supersonic metal cluster beams of refractory metals: Spectral investigations of ultracold Mo_2_. *J Chem Phys* 1983; **78**: 1627-37.

5. Castleman, AW, Jr., Keesee, RG. Clusters: Bridging the gas and condensed phases. *Acc Chem Res* 1986; **19**: 413-9.

6. Leuchtner, RE, Harms, AC, Castleman, AW, Jr. Thermal metal cluster anion reactions: Behavior of aluminum clusters with oxygen. *J Chem Phys* 1989; **91**: 2753-4.

7. Zimmerman, JA, Eyler, JR, Bach, SBH *et al.* "Magic number" carbon clusters: Ionization potentials and selective reactivity. *J Chem Phys* 1991; **94**: 3556-62.

8. Wang, LS, Cheng, HS, Fan, J. Photoelectron spectroscopy of size‐selected transition metal clusters: Fe_n_^-^, n=3–24. *J Chem Phys* 1995; **102**: 9480-93.

9. Duncan, MA. Invited review article: Laser vaporization cluster sources. *Rev Sci Instrum* 2012; **83**: 041101.

10. Adams, NG, Smith, D. The selected ion flow tube (SIFT): A technique for studying ion-neutral reactions. *Int J Mass Spectrom* 1976; **21**: 349-59.

11. Eller, K, Schwarz, H. Organometallic chemistry in the gas-phase. *Chem Rev* 1991; **91**: 1121-77.

12. Schwarz, H. Doping effects in cluster-mediated bond activation. *Angew Chem Int Ed* 2015; **54**: 10090-100100.

13. Veldeman, N, Lievens, P, Andersson, M. Size-dependent carbon monoxide adsorption on neutral gold clusters. *J Phys Chem A* 2005; **109**: 11793-801.

14. Roach, PJ, Woodward, WH, Castleman, AW, Jr. *et al.* Complementary active sites cause size-selective reactivity of aluminum cluster anions with water. *Science* 2009; **323**: 492-5.

15. De Haeck, J, Veldeman, N, Claes, P *et al.* Carbon monoxide adsorption on silver doped gold clusters. *J Phys Chem A* 2011; **115**: 2103-9.

16. Le, HT, Lang, SM, De Haeck, J *et al.* Carbon monoxide adsorption on neutral and cationic vanadium doped gold clusters. *Phys Chem Chem Phys* 2012; **14**: 9350-8.

17. Kerpal, C, Harding, DJ, Rayner, DM *et al.* Small platinum cluster hydrides in the gas phase. *J Phys Chem A* 2013; **117**: 8230-7.

18. Janssens, E, Le, HT, Lievens, P. Adsorption of propene on neutral gold clusters in the gas phase. *Chem- Eur J* 2015; **21**: 15256-62.

19. Dong, F, Heinbuch, S, Xie, Y *et al.* C=C bond cleavage on neutral VO_3_(V_2_O_5_)_n_ clusters. *J Am Chem Soc* 2009; **131**: 1057-66.

20. Ferrari, P, Vanbuel, J, Janssens, E *et al.* Tuning the reactivity of small metal clusters by heteroatom doping. *Acc Chem Res* 2018; **51**: 3174-82.

21. Yuan, C, Liu, X, Zeng, C *et al.* All-solid-state deep ultraviolet laser for single-photon ionization mass spectrometry. *Rev Sci Instrum* 2016; **87**: 024102.

22. Luo, Z. Deep ultraviolet single‐photon ionization mass spectrometry. In: Aliofkhazraei, M (ed.) *Mass Spectrometry*. Rijeka: InTech, 2017, 3-21.

23. Chen, J, Luo, Z, Fu, H *et al.* Photoreactions of porphyrins initiated by deep ultraviolet single photons. *J Phys Chem A* 2017; **121**: 4626-32.

24. Wu, H, Yuan, C, Zhang, H *et al.* Ultrafast deep-ultraviolet laser ionization mass spectrometry applicable to identify phenylenediamine isomers. *Anal Chem* 2018; **90**: 10635-40.

25. Wu, H, Guo, M, Yang, M *et al.* Selective C-C and C-N bond activation in dopamine and norepinephrine under deep ultraviolet laser irradiation. *Chem Commun* 2019; **55**: 4015-8.

26. Zhang, H, Wu, H, Jia, Y *et al.* An integrated instrument of DUV-IR photoionization mass spectrometry and spectroscopy for neutral clusters. *Rev Sci Instrum* 2019; **90**: 073101.

27. Zhang, H, Wu, H, Geng, L *et al.* Furthering the reaction mechanism of cationic vanadium clusters towards oxygen. *Phys Chem Chem Phys* 2019; **21**: 11234-41.

28. Hansen, K, Ferrari, P. Influence of thermal radiation on hot cluster decay rates and abundances. *Chin J Chem Phys* 2019; **32**: 167-74.

29. Dong, F, Heinbuch, S, He, SG *et al.* Formation and distribution of neutral vanadium, niobium, and tantalum oxide clusters: Single photon ionization at 26.5 eV. *J Chem Phys* 2006; **125**: 164318.

30. Li, Y, Qi, F. Recent applications of synchrotron VUV photoionization mass spectrometry: Insight into combustion chemistry. *Acc Chem Res* 2009; **43**: 68-78.

31. Hua, L, Wu, Q, Hou, K *et al.* Single photon ionization and chemical ionization combined ion source based on a vacuum ultraviolet lamp for orthogonal acceleration time-of-flight mass spectrometry. *Anal Chem* 2011; **83**: 5309-16.

32. Schlappi, B, Litman, JH, Ferreiro, JJ *et al.* A pulsed uniform Laval expansion coupled with single photon ionization and mass spectrometric detection for the study of large molecular aggregates. *Phys Chem Chem Phys* 2015; **17**: 25761-71.

33. Lv, J, Wang, Y, Zhu, L *et al.* Particle-swarm structure prediction on clusters. *J Chem Phys* 2012; **137**: 084104.

34. Wang, Y, Lv, J, Zhu, L *et al.* CALYPSO: A method for crystal structure prediction. *Comput Phys Commun* 2012; **183**: 2063-70.

35. Sun, WG, Wang, JJ, Lu, C *et al.* Evolution of the structural and electronic properties of medium-sized sodium clusters: a honeycomb-like Na_20_ cluster. *Inorg Chem* 2017; **56**: 1241-8.

36. Tian, Y, Wei, D, Jin, Y *et al.* Exhaustive exploration of MgB_n_ (n = 10-20) clusters and their anions. *Phys Chem Chem Phys* 2019; **21**: 6935-41.

37. Ren, M, Jin, S, Wei, D *et al.* NbB_12_^-^: A new member of half-sandwich type doped boron clusters with high stability. *Phys Chem Chem Phys* 2019; **21**: 21746-52.

38. Sun, W, Xia, X, Lu, C *et al.* Probing the structural and electronic properties of zirconium doped boron clusters: Zr distorted B_12_ ligand framework. *Phys Chem Chem Phys* 2018; **20**: 23740-6.

39. Le Chen, B, Sun, WG, Kuang, XY *et al.* Structural stability and evolution of medium-sized tantalum-doped boron clusters: A half-sandwich-structured TaB_12_^-^ cluster. *Inorg Chem* 2018; **57**: 343-50.

40. Alonso-Lanza, T, Ayuela, A, Aguilera-Granja, F. Chemical bonding of transition-metal Co_13_ clusters with graphene. *ChemPhysChem* 2015; **16**: 3700-10.

41. Anderson, ML, Lacz, A, Drewello, T *et al.* The chemistry of nitrogen oxides on small size-selected cobalt clusters, Co_n_^+^. *J Chem Phys* 2009; **130**: 064305.

42. Morse, MD. Clusters of transition-metal atoms. *Chem Rev* 1986; **86**: 1049-109.

43. McCool, NS, Robinson, DM, Sheats, JE *et al.* A Co_4_O_4_ "cubane" water oxidation catalyst inspired by photosynthesis. *J Am Chem Soc* 2011; **133**: 11446-9.

44. Sebetci, A. Cobalt clusters (Co_n_, n ≤ 6) and their anions. *Chem Phys* 2008; **354**: 196-201.

45. Pakiari, AH, Dehghanpisheh, E. The electronic structure of nanoparticle: Theoretical study of small cobalt clusters (Co_n_ , n = 2–5) (part A). *Struct Chem* 2015; **27**: 583-93.

46. Li, Z-q, Gu, B-l. Electronic-structure calculations of cobalt clusters. *Phys Rev B* 1993; **47**: 13611-4.

47. Reuse, FA, Khanna, SN. Geometry, electronic structure and magnetism of small Co_n_ (n=2-8) clusters. *Chem Phys Lett* 1995; **234**: 77-81.

48. Jones, NO, Beltran, MR, Khanna, SN *et al.* Hydrogen adsorption and magnetic behavior of Fe_n_ and Co_n_ clusters: Controlling the magnetic moment and anisotropy one atom at a time. *Phys Rev B* 2004; **70**: 165406.

49. Cui, J, Zhou, X, Wang, G *et al.* Infrared photodissociation spectroscopy of mass-selected homoleptic cobalt carbonyl cluster cations in the gas phase. *J Phys Chem A* 2014; **118**: 2719-27.

50. Tombers, M, Barzen, L, Niedner-Schatteburg, G. Inverse H/D isotope effects in benzene activation by cationic and anionic cobalt clusters. *J Phys Chem A* 2013; **117**: 1197-203.

51. Lu, S, Zhang, J, Duan, H. Melting behaviors of Co_N_ (N=13, 14, 38, 55, 56) clusters. *Chem Phys* 2009; **363**: 7-12.

52. Tomihara, R, Koyasu, K, Tsukuda, T. Observation and the origin of magic compositions of Co_n_O_m_^–^ formed in oxidation of cobalt cluster anions. *J Phys Chem C* 2017; **121**: 10957-63.

53. Mattioli, G, Giannozzi, P, Amore Bonapasta, A *et al.* Reaction pathways for oxygen evolution promoted by cobalt catalyst. *J Am Chem Soc* 2013; **135**: 15353-63.

54. Rodríguez-López, JL, Aguilera-Granja, F, Michaelian, K *et al.* Structure and magnetism of cobalt clusters. *Phys Rev B* 2003; **67**: 174413.

55. van Dijk, CN, Roy, DR, Fielicke, A *et al.* Structure investigation of Co_x_O_y_^+^ (x=3–6, y=3–8) clusters by IR vibrational spectroscopy and DFT calculations. *Eur Phys J D* 2014; **68**: 357.

56. Datta, S, Kabir, M, Ganguly, S *et al.* Structure, bonding, and magnetism of cobalt clusters from first-principles calculations. *Phys Rev B* 2007; **76**: 014429.

57. Castro, M, Jamorski, C, Salahub, DR. Structure, bonding, and magnetism of small Fe_n_, Co_n_, and Ni_n_ clusters, n ≤ 5. *Chem Phys Lett* 1997; **271**: 133-42.

58. Li, X, Siegbahn, PEM. Water oxidation mechanism for synthetic Co-oxides with small nuclearity. *J Am Chem Soc* 2013; **135**: 13804-13.

59. Jena, P, Castleman, AW, Jr. Clusters: A bridge across the disciplines of physics and chemistry. *Proc Natl Acad Sci U S A* 2006; **103**: 10560-9.

60. Jena, P, Sun, Q. Super atomic clusters: Design rules and potential for building blocks of materials. *Chem Rev* 2018; **118**: 5755-80.

61. Nößler, M, Mitrić, R, Bonačić-Koutecký, V *et al.* Generation of oxygen radical centers in binary neutral metal oxide clusters for catalytic oxidation reactions. *Angew Chem Int Ed* 2010; **49**: 407-10.

62. Dibble, CJ, Akin, ST, Ard, S *et al.* Photodissociation of cobalt and nickel oxide cluster cations. *J Phys Chem A* 2012; **116**: 5398-404.

63. Vajda, S, Pellin, MJ, Greeley, JP *et al.* Subnanometre platinum clusters as highly active and selective catalysts for the oxidative dehydrogenation of propane. *Nat Mater* 2009; **8**: 213-6.

64. Ota, K, Koyasu, K, Ohshimo, K *et al.* Structures of cobalt oxide cluster cations studied by ion mobility mass spectrometry. *Chem Phys Lett* 2013; **588**: 63-7.

65. Liu, F, Li, FX, Armentrout, PB. Guided ion-beam studies of the reactions of Co_n_^+^ (n=2-20) with O_2_: Cobalt cluster-oxide and -dioxide bond energies. *J Chem Phys* 2005; **123**: 64304.

66. Lee, S, Halder, A, Ferguson, GA *et al.* Subnanometer cobalt oxide clusters as selective low temperature oxidative dehydrogenation catalysts. *Nat commun* 2019; **10**: 954.

67. Yin, S, Xue, W, Ding, X-L *et al.* Formation, distribution, and structures of oxygen-rich iron and cobalt oxide clusters. *Int J Mass Spectrom* 2009; **281**: 72-8.

68. Aguilera-del-Toro, RH, Aguilera-Granja, F, Vega, A *et al.* Structure, fragmentation patterns, and magnetic properties of small cobalt oxide clusters. *Phys Chem Chem Phys* 2014; **16**: 21732-41.

69. Wang, Q, Sun, Q, Sakurai, M *et al.* Geometry and electronic structure of magic iron oxide clusters. *Phys Rev B* 1999; **59**: 12672-7.

70. Sun, Q, Wang, Q, Parlinski, K *et al.* First-principles studies on the intrinsic stability of the magic Fe_13_O_8_ cluster. *Phys Rev B* 2000; **61**: 5781-5.

71. Kortus, J, Pederson, MR. Magnetic and vibrational properties of the uniaxial Fe_13_O_8_ cluster. *Phys Rev B* 2000; **62**: 5755-9.

72. Zhao, Y, Chen, X, Li, J. TGMin: A global-minimum structure search program based on a constrained basin-hopping algorithm. *Nano Res* 2017; **10**: 3407-20.

73. Anisimov, VV, Zaanen, J, Andersen, OK. Band theory and Mott insulators: Hubbard U instead of Stoner I. *Phys Rev B Condens Matter* 1991; **44**: 943-54.

74. Wang, L, Maxisch, T, Ceder, G. Oxidation energies of transition metal oxides within the GGA+U framework. *Phys Rev B* 2006; **73**: 195107.

75. Kresse, G, Hafner, J. Ab initio molecular dynamics for liquid metals. *Phys Rev B* 1993; **47**: 558-61.

76. Binggeli, N, Chelikowsky, JR. Langevin molecular dynamics with quantum forces: Application to silicon clusters. *Phys Rev B* 1994; **50**: 11764-70.

77. Frisch, MJ, Trucks, GW, Schlegel, HB *et al.* Gaussian 16 Revision C.01. Wallingford CT: Gaussian, Inc.; 2016.

78. Tyo, EC, Nossler, M, Harmon, CL *et al.* Investigating reactive superoxide units bound to zirconium oxide cations. *J Phys Chem C* 2011; **115**: 21559-66.

79. Pal, R, Wang, LM, Pei, Y *et al.* Unraveling the mechanisms of O_2_ activation by size-selected gold clusters: Transition from superoxo to peroxo chemisorption. *J Am Chem Soc* 2012; **134**: 9438-45.

80. Feyel., S, bler., JD, ckendorf., RH *et al.* Activation of methane by oligomeric (Al_2_O_3_)_x_^+^ (x=3,4,5): The role of oxygen-centered radicals in thermal hydrogen-atom abstraction. *Angew Chem Int Ed* 2008; **47**: 1946-50.

81. Chen, J-J, Li, XN, Chen, Q *et al.* Neutral Au_1_-doped cluster catalysts AuTi_2_O_3-6_ for CO oxidation by O_2_. *J Am Chem Soc* 2019; **141**: 2027-34.

82. Velde, GT, Bickelhaupt, FM, Baerends, EJ *et al.* Chemistry with ADF. *J Comput Chem* 2001; **22**: 931-67.

83. Fonseca Guerra, C, Snijders, JG, te Velde, G *et al.* Towards an order- N DFT method. *Theoretical Chemistry Accounts: Theory, Computation, and Modeling (Theoretica Chimica Acta)* 1998; **99**: 391-403.

84. Baerends, EJ, Ziegler, T, Autschbach, J *et al.* *ADF2018, SCM, Theoretical Chemistry*. <http://www.scm.com>

85. Perdew, JP, Burke, K, Ernzerhof, M. Generalized gradient approximation made simple. *Phys Rev Lett* 1996; **77**: 3865-8.

86. Zhao, Y, Truhlar, DG. A new local density functional for main-group thermochemistry, transition metal bonding, thermochemical kinetics, and noncovalent interactions. *J Chem Phys* 2006; **125**: 194101.

87. Zhao, Y, Truhlar, DG. The M06 suite of density functionals for main group thermochemistry, thermochemical kinetics, noncovalent interactions, excited states,and transition elements two new functionals and systematic testingof four M06-class functionals and 12 other functionals. *Theor Chem Acc* 2008; **120**: 215-41.

88. Lenthe, EV, Baerends, EJ. Optimized Slater-type basis sets for the elements 1–118. *J Comput Chem* 2003; **24**: 1142-56.

89. Grimme, S, Antony, J, Ehrlich, S *et al.* Relativistic regular two-component Hamiltonians. *J Chem Phys* 1993; **99**: 4597-610.

90. Mulliken, RS. Electronic population analysis on LCAO‐MO molecular wave functions. III. Effects of hybridization on overlap and gross ao populations. *J Chem Phys* 1955; **23**: 2338-42.

91. Hirshfeld, FL. Bonded-atom fragments for describing molecular charge densities. *Theoret Clim Acta (Berl)* 1977; **44**: 129-38.

92. Bickelhaupt, FM, Hommes, NJRvE, Guerra, CF *et al.* The carbon−lithium electron pair bond in (CH_3_Li)_n_ (n = 1, 2, 4). *Organometallics* 1996; **15**: 2923-31.

93. Swart, M, Vanduijnen, PT, Snijders, JG. A charge analysis derived from an atomic multipole expansion. *J Comput Chem* 2001; **22**: 79-88.

94. Elser, V, Haddon, RC. Icosahedral C_60_: An aromatic molecule with a vanishingly small ring current magnetic susceptibility. *Nature* 1987; **325**: 792-4.

95. Schleyer, PvR, Maerker, C, Dransfeld, A *et al.* Nucleus-independent chemical shifts: A simple and efficient aromaticity probe. *J Am Chem Soc* 1996; **118**: 6317-8.

96. Chen, Z, Corminboeu, C, Heine, T *et al.* Do all-metal antiaromatic clusters exist. *J Am Chem Soc* 2003; **125**: 13930-1.

97. Chen, ZF, Wannere, CS, Corminboeuf, C *et al.* Nucleus-independent chemical shifts (NICS) as an aromaticity criterion. *Chem Rev* 2005; **105**: 3842-88.

98. Cui, P, Hu, HS, Zhao, B *et al.* A multicentre-bonded [Zn^(I)^]_8_ cluster with cubic aromaticity. *Nat Commun* 2015; **6**: 6331.

99. Lu, T, Chen, F. Multiwfn: A multifunctional wavefunction analyzer. *J Comput Chem* 2012; **33**: 580-92.

100. Humphrey, W, Dalke, A, Schulten, K. VMD: Visual molecular dynamics. *J Molec Graphics* 1996; **14**: 33-8.

101. Johnson, GE, Reveles, JU, Reilly, NM *et al.* Influence of stoichiometry and charge state on the structure and reactivity of cobalt oxide clusters with CO. *J Phys Chem A* 2008; **112**: 11330-40.

102. Jusélius, J, Sundholm, D, Gauss, J. Calculation of current densities using gauge-including atomic orbitals. *J Chem Phys* 2004; **121**: 3952-63.

103. Fliegl, H, Taubert, S, Lehtonen, O *et al.* The gauge including magnetically induced current method. *Phys Chem Chem Phys* 2011; **13**: 20500-18.
